# Plasma proteomics defines two reproducible subphenotypes of sepsis-associated acute kidney injury with distinct outcomes

**DOI:** 10.1038/s41467-026-76889-3

**Published:** 2026-08-19

**Authors:** Matthieu Legrand, Hanh Nguyen, Carolyn S. Calfee, Yingying Lu, Michael Harhay, Kathleen Liu, David Chen, Juliane Bernholz, Michael Matthay, Alexandre Mebazaa, Peter Pickkers, Ernesto Rojas, Alexendar R. Perez

**Affiliations:** 1 Department of Anesthesia and Perioperative Care, Division of Critical Care Medicine, University of California San Francisco, San Francisco, CA USA; 2 French Clinical Research Infrastructure Network Initiative–Cardio Renal Clinical Trialists Network, Nancy, France; 3 Department of Medicine and Anesthesia, Division of Pulmonary, Critical Care Medicine, University of California, San Francisco, San Francisco, CA USA; 4 Department of Biostatistics, Epidemiology and Informatics, Perelman School of Medicine, University of Pennsylvania, Philadelphia, PA USA; 5 Palliative and Advanced Illness Research (PAIR) Center, University of Pennsylvania, Philadelphia, PA USA; 6 Department of Medicine, Division of Nephrology, University of California, San Francisco, San Francisco, CA USA; 7 AM-Pharma B.V, Utrecht, the Netherlands; 8 Département d’Anesthésie-Réanimation, Hôpital Lariboisière, Assistance Publique–Hôpitaux de Paris (AP-HP), Université Paris Cité, Paris, France; 9 Department of Intensive Care Medicine, Radboud University Medical Center, Nijmegen, the Netherlands

**Keywords:** Predictive markers, Acute kidney injury, Outcomes research

## Abstract

Sepsis is the leading cause of acute kidney injury in critically ill patients, and this complication carries a high risk of death. The biology underlying it varies between patients, which may explain why treatments have not succeeded. Here we show, using an ensemble method that groups patients by patterns across hundreds of blood proteins in three independent groups of patients, that sepsis-associated acute kidney injury comprises two reproducible subphenotypes. One subphenotype shows widespread activation of inflammation, metabolism, and oxidative stress; the other shows a quieter profile. Patients in the inflammatory subtype have higher mortality, more complications, and fewer days alive and out of the intensive care unit. A simple seven-protein test reproduces these groupings and identifies them accurately in separate cohorts. In a randomized trial, the drug ilofotase alfa appears to benefit the inflammatory subtype but not the other, suggesting that this classification could guide future treatment.

## Abstract (web summary)

Sepsis is a leading cause of acute kidney injury in critically ill patients and carries a high risk of death, yet its biology varies between patients. Here, the authors show that plasma proteomics defines two subtypes with distinct outcomes and drug response, identified by a seven-protein test.

## Introduction

Sepsis-associated acute kidney injury (SAKI) is a frequent complication in critically ill patients^1,2^. It affects upwards of 50% of septic patients with significant impacts on remote organ damage (e.g., heart, lung), prolonged hospitalization, risk of chronic kidney disease, and overall survival^3^. Therapeutic interventions for SAKI remain largely empirical, non-specific, and supportive^4^ and result from a limited understanding of its underlying complex pathophysiology. Current definitions of acute kidney injury (AKI) do not capture the underlying biological heterogeneity of clinically septic patients, nor do they effectively guide therapeutic decision-making^5^.

Recent evidence suggests that SAKI reflects a spectrum of distinct biological and clinical subtypes, or subphenotypes, that may differ in their mechanisms of injury, risk of adverse outcomes, and response to interventions^1,3^. Phenotyping—defined as the identification of biologically meaningful patient subgroups within a syndrome—has the potential to refine clinical classification, enhance prognostic accuracy, and enable precision medicine approaches in critical care. In acute critical illnesses such as sepsis and acute respiratory distress syndrome (ARDS)^6,7^, clustering approaches have revealed reproducible subphenotypes with distinct inflammatory profiles and differential treatment responses^6,8,9^. However, similar efforts in SAKI remain elusive. Among patients with sepsis, those developing SAKI have worse outcomes compared to those who do not develop SAKI, justifying the need to specifically investigate this population.

In this study, we sought to identify subphenotypes of patients with sepsis-associated acute kidney injury (SAKI) using unsupervised methods with high-dimensional proteomic data. The aim was to identify SAKI subphenotypes and what, if any, association existed between those subphenotypes and clinical outcomes (mortality at 28 and 90 Days, major adverse kidney events). A secondary aim explored potential heterogeneity in therapeutic efficacy to recombinant alkaline phosphatase (ilofotase alfa), a treatment aimed to improve renal function in critical illness. By uncovering biomarker-based clusters, using data-driven methods in patients with SAKI, the results of this study may inform future development of subphenotype-targeted therapies and improve the design of clinical trials in this high-risk population.

## Results

### Ensemble method-derived subphenotype discovery

**Fig. 1:**
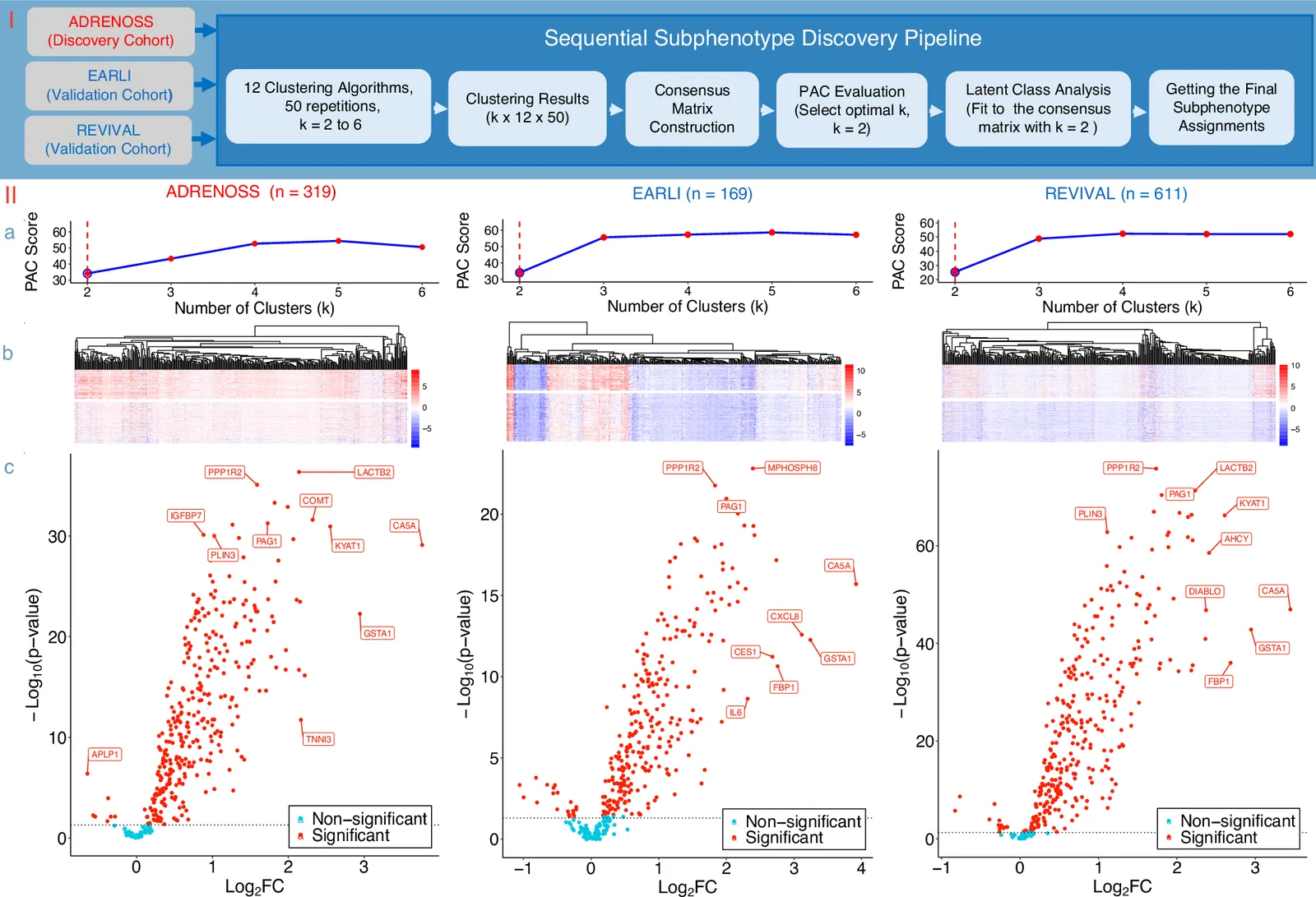
Sequential subphenotype discovery pipeline and differential expression analysis. Part I: Sequential subphenotype discovery pipeline. Ensemble consensus clustering was performed using 12 algorithms with 50 repetitions (*k* = 2–6). The optimal cluster number was selected by PAC, identifying *k* = 2 as optimal. Final subphenotype assignments were derived using latent class analysis fitted to the consensus matrix, applied in ADRENOSS (*n* = 319), EARLI (*n* = 169), and REVIVAL (*n* = 611). Part II: Subphenotype identification and differential protein expression across three cohorts. **a** PAC scores for *k* = 2–6; dashed red line indicates *k* = 2, the most stable partition. **b** Heatmap of Olink NPX protein expression values. Proteins (*x*-axis) and patients (*y*-axis) grouped by subphenotype (Subphenotype 1, upper; Subphenotype 2, lower). **c** Volcano plot of differential protein expression. Positive Log₂FC = upregulated in Subphenotype 1; negative Log₂FC = upregulated in Subphenotype 2. Significant proteins (BH-adjusted *p*-value < 0.05, |Log₂FC| > 1) are shown in red; non-significant in cyan. Wilcoxon rank-sum test, two-sided, with Benjamini–Hochberg correction.

Three hundred and nineteen patients from the ADRENOSS cohort met the definition of early SAKI and formed the discovery cohort. Using an ensemble clustering approach, two distinct subphenotypes were identified. Subphenotype 1 included 132 patients, and Subphenotype 2 included 187 patients. Baseline characteristics of patients by cluster are detailed in Table 1. Principal component analysis (PCA) demonstrated a clear separation between the two clusters, indicating robust structural differences in the proteomic signatures (Fig. 1—Part II). These subphenotypes displayed distinct biological profiles, further characterized by differential protein expression and pathway enrichment analyses.

**Table 1:** Characteristics of the patients at inclusion in ADRENOS, EARLI, and REVIVAL

Patients characteristic	ADRENOSS				EARLI				REVIVAL			
	Sub. 1	Sub. 2	*P*-value^b^	SMD^c^	Sub. 1	Sub. 2	*P*-value^b^	SMD^c^	Sub. 1	Sub. 2	*P*-value^b^	SMD^c^
	*N* = 132^a^	*N* = 187^a^			*N* = 59^a^	*N* = 110^a^			*N* = 275^a^	*N* = 336^a^		
**Age (years)**	66.2 (14.7)	68.0 (13.0)	0.3	−0.13	64.7 (15.9)	69.9 (15.8)	0.045	−0.33	66.7 (11.7)	68.8 (11.4)	0.03	−0.18
**Sex (Male)**	74 (56%)	118 (63%)	0.3	0.14	35 (59%)	59 (54%)	0.6	−0.11	160 (58%)	228 (68%)	0.017	−0.21
**Comorbidities**												
Hypertension, *n*(%)	71 (55%)	110 (59%)	0.6	0.08	16 (27%)	67 (61%)	<0.001	−0.72	88 (32%)	117 (35%)	0.5	−0.06
COPD, *n*(%)	18 (14%)	34 (18%)	0.4	−0.12	6 (10%)	17 (15%)	0.5	−0.16	9 (3%)	35 (10%)	0.001	−0.29
Diabetes mellitus, *n*(%)	47 (36%)	57 (30%)	0.4	0.11	12 (20%)	36 (33%)	0.13	−0.29	35 (13%)	40 (12%)	0.9	0.02
Coronary artery disease, *n*(%)	16 (12%)	32 (17%)	0.3	−0.14	1 (2%)	23 (21%)	0.001	−0.63	10 (4%)	10 (3%)	0.8	0.04
Peripheral artery disease, *n*(%)	16 (13%)	23 (12%)	>0.9	0.01	0 (0%)	5 (5%)	0.2	−0.31	7 (3%)	6 (2%)	0.7	0.05
Chronic heart failure, *n*(%)	17 (13%)	23 (12%)		0.03	12 (20%)	31 (28%)	0.4	−0.18	3 (1%)	3 (1%)	>0.9	0.02
Chronic liver disease, *n*(%)	8 (6%)	11 (6%)	>0.9	0.01	10 (17%)	11 (10%)	0.3	0.2	5 (2%)	8 (2%)	0.8	−0.04
Chronic kidney disease, *n*(%)	24 (19%)	30 (16%)	0.7	0.07	9 (15%)	31 (28%)	0.09	−0.32	15 (5%)	17 (5%)	>0.9	0.02
BMI (kg/m²)	28.0 (7.6)	28.0 (7.2)	>0.9	0	26.3 (8.4)	29.0 (9.6)	0.085	−0.29	30.4 (8.2)	29.7 (8.3)	0.3	0.08
**Sepsis characteristics**												
RRT at enrollment	26 (20%)	9(5%)	<0.001	0.44	13(22%)	3(3%)	<0.001	0.61		–		
Serum creatinine at enrollment (mg/dL)	2.8 [2.0– 4.0]	1.8 [1.4– 2.8]	<0.001	0.39	2.2 [1.7 –3.2]	1.7 [1.3– 2.8]	0.14	0.26	2.5 [1.8– 3.3]	1.8 [1.4– 2.5]	<0.001	0.56
Pre-AKI serum creatinine (mg/dL)	1.0 [0.8– 1.1]	1.0 [0.8– 1.0]	0.5	0.08	1.3 [0.9–2.6]	1.1 [0.8– 1.6]	0.12	0.50	0.9 [0.8– 1.1]	1.0 [0.8– 1.1]	0.7	0.03
Pre AKI eGFR (mL/min/1.73 m^2)^	78.3 [75.9– 80.8]	77.9 [76.1–80.5]	0.2	−0.16	55.0 [24.1– 88.1]	66.2 [40.9– 85.1]	0.3	−0.29	74.6 [60.2– 88.3]	74.2 [60.6– 89.9]	>0.9	0.00
Heart rate (bpm)	108 [94–123]	99 [87–116]	0.007	0.32	123 [107–139]	108 [97–125]	0.002	0.53	117 [92–133]	109 [85–124]	0.009	0.21
Mean blood pressure (mmHg)	72 [60– 84]	72 [62–88]	0.5	−0.08	102 [88–114]	99 [93–115]	0.6	−0.09	60 [52– 66]	60 [54–68]	0.2	−0.11
Respiratory rate (breaths/min)	22 [18–27]	22 [18–27]	0.6	0.05	35 [33– 38]	35 [30–40]	0.3	0.19	26 [20– 33]	25 [19–30]	0.009	0.21
SOFA score	10.0 [8.0– 12.0]	7.0 [5.0– 10.0]	<0.001	0.71					10.3 (2.5)	8.4 (1.7)	<0.001	0.91
APACHE II score	19.0 [15.0– 23.0]	16.0 [12.0– 20.0]	<0.001	0.45	39.1 (8.8)	28.4 (8.2)	<0.001	1.26	25.1 (7.3)	21.9 (7.0)	<0.001	0.44
Lactate (mmol/L)	4.0 [2.5– 7.2]	2.3 [1.4– 3.6]	<0.001	0.65	6.9 (4.8)	4.6 (4.3)	0.021	0.5	4.1 (3.0)	2.2 (1.4)	<0.001	0.82
**Sources of sepsis (Y)**			0.031	0.3			0.2	0.22			0.059	−0.01
Lung	33 (25%)	68 (36%)			29 (49%)	67 (61%)			67 (24%)	108 (32%)		
Blood stream	27 (20%)	38 (20%)			–	–						
Urinary tract	16 (12%)	25 (13%)			–	–			33 (12%)	24 (7%)		
Catheter	12 (9%)	4 (2%)			–	–						
Abdominal	7 (5%)	12 (6%)			–	–			107 (39%)	120 (36%)		
Endocarditis or other	37 (28%)	40 (21%)			30 (51%)	43 (39%)			68 (25%)	84 (25%)		
**Mechanical ventilation,** *n* (%)	68 (52%)	64 (34%)	0.008	−0.19	46 (82%)	62 (65%)	0.034	0.4	229 (83%)	236 (70%)		0.27
**Norepinephrine,** *n* (%)	107 (81%)	119 (64%)	0.001	0.34	50 (96%)	69 (95%)	>0.9	0.08	238 (87%)	286 (85%)	0.7	0.04

*Sub* subphenotype, *COPD* chronic obstructive pulmonary disease, *BMI* body mass index, *SOFA* sequential organ failure assessment, *APACHE II* Acute Physiology And Chronic Health Evaluation II, *RRT* renal replacement therapy.

^a^Mean (SD); Median [Q1–Q3]; *n* (%).

^b^Two-sided Welch two-sample *t*-test for continuous variables; Fisher’s Exact Test when any expected cell count < 5, Pearson’s Chi-squared test otherwise, for categorical variables. No adjustment for multiple comparisons was applied to *p*-values.

^c^Standardized mean difference.

### Ensemble method-derived subphenotype validation

**Fig. 2:**
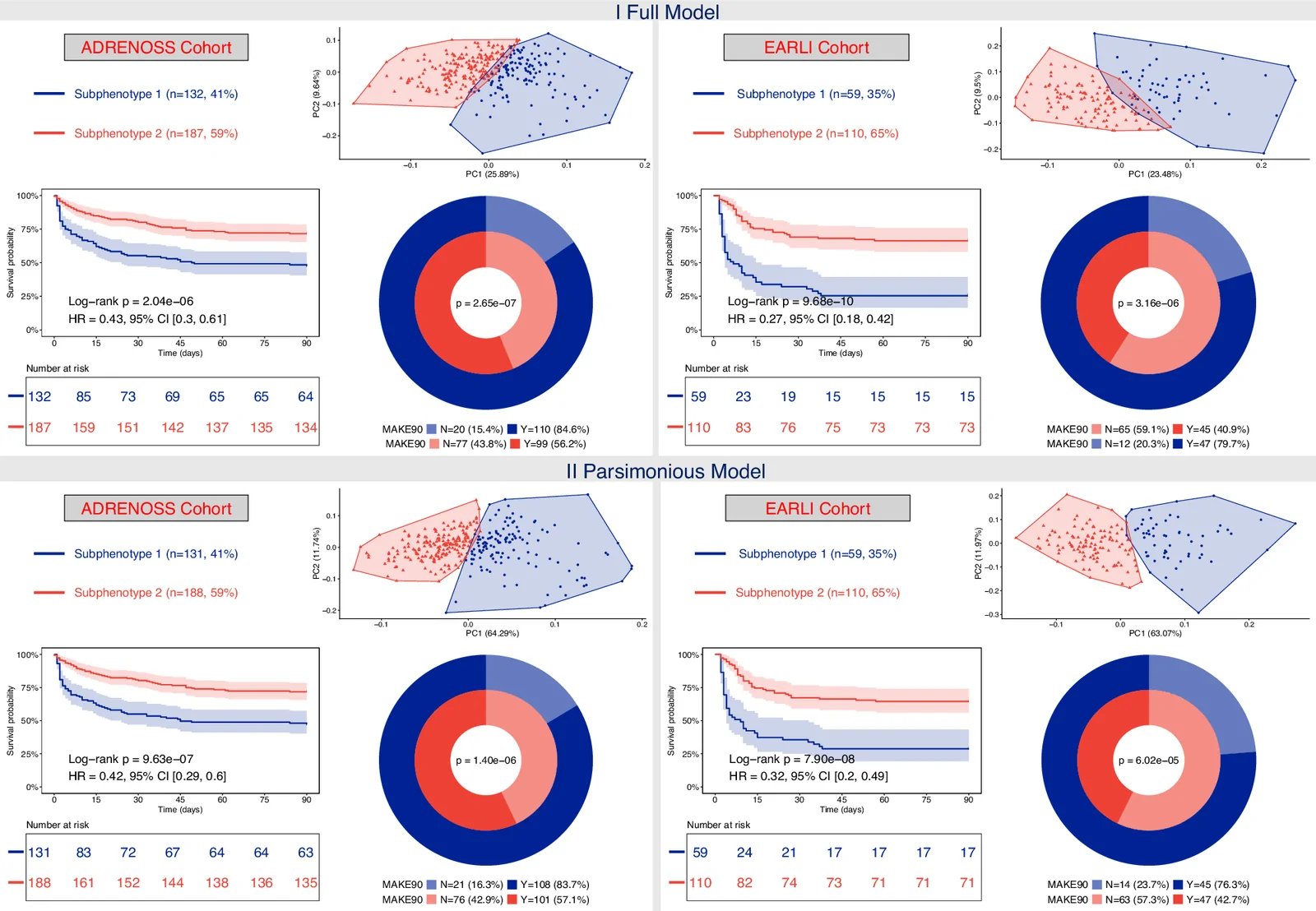
Subphenotype comparison between full and parsimonious models in ADRENOSS and EARLI cohorts. Subphenotypes derived from the full biomarker model (Part I) and the parsimonious 7-protein model (Part II) are compared in ADRENOSS and EARLI. For each cohort, principal component analysis (PCA) plots show proteomic separation between subphenotypes, Kaplan–Meier curves show 90-day survival probability, and doughnut charts show the distribution of Major Adverse Kidney Events at 90 days (MAKE90; N = no event, Y = event) by subphenotype, with the outer ring representing Subphenotype 1 and the inner ring representing Subphenotype 2. Shaded bands represent 95% confidence intervals. Numbers at risk are displayed below each Kaplan–Meier curve. Hazard ratios (HR) with 95% confidence intervals were estimated using Cox proportional hazards regression. Survival differences between subphenotypes were assessed using two-sided log-rank tests. MAKE90 differences were assessed using two-sided *χ*^2^ tests. No adjustments were made for multiple comparisons. Exact *p*-values are displayed within each panel. In ADRENOSS, the full biomarker model identified Subphenotype 1 (*n* = 132, 41%) and Subphenotype 2 (*n* = 187, 59%); log-rank *p* = 2.04 × 10^-6^, HR = 0.43 (95% CI [0.30, 0.61]); MAKE90 *χ*^2^ *p* = 2.65 × 10^−7^. The parsimonious model identified Subphenotype 1 (*n* = 131, 41%) and Subphenotype 2 (*n* = 188, 59%); log-rank *p* = 9.63 × 10^−7^, HR = 0.42 (95% CI [0.29, 0.60]); MAKE90 *χ*^2^ *p* = 1.40 × 10^−6^. In EARLI, the full biomarker model identified Subphenotype 1 (*n* = 59, 35%) and Subphenotype 2 (*n* = 110, 65%); log-rank *p* = 7.90 × 10^−8^, HR = 0.32 (95% CI [0.20, 0.49]); MAKE90 *χ*^2^ *p* = 6.02 × 10^−5^. The parsimonious model identified Subphenotype 1 (*n* = 59, 35%) and Subphenotype 2 (*n* = 110, 65%); log-rank *p* = 9.68 × 10^−10^, HR = 0.27 (95% CI [0.18, 0.42]); MAKE90 *χ*^2^ *p* = 3.16 × 10^−6^.

Validation was performed in 169 patients from the EARLI cohort and 611 patients from the REVIVAL trial. The EARLI study originally enrolled 1622 patients; after restricting to participants with available Olink biomarker data, acute kidney injury present, and no end-stage renal disease, 169 patients were retained for validation (Supplementary Fig. 1). The REVIVAL trial originally reported 649 patients, of whom 611 had available Olink biomarker measurements. The EARLI cohort included data for all 366 proteins, while the REVIVAL cohort included 359 proteins due to missing NPX values for seven proteins. Applying the same ensemble clustering pipeline, two consistent subphenotypes were identified in both validation cohorts, with PCA plots showing clear separation between clusters and PC1 explaining a substantial proportion of the variance (Fig. 2—Part I and Supplementary Fig. 2—Part a). Subphenotypes were identified in both validation cohorts (Fig. 2).

Rankings based on variable importance from the nearest shrunken centroid (NSC) method and differential expression statistics were highly correlated across cohorts (Supplementary Table 1 and Supplementary Fig. 3). Among the top 10% proteins identified in each cohort, 24 proteins were consistently shared across ADRENOSS, EARLI, and REVIVAL, confirming the reproducibility of the clustering structure.

### Differential proteomic expression and pathway enrichment

PCA revealed a marked separation between the subphenotypes. Among the 321 proteins significantly differentially expressed (adjusted *p* < 0.05), 98% were upregulated in Subphenotype 1 compared with Subphenotype 2 (Fig. 1, part II). Across cohorts, Subphenotype 1 was characterized by a metabolically active, pro-inflammatory, and oxidative stress–enriched profile. Elevated proteins included markers of oxidative stress regulation, such as superoxide dismutase (Cu–Zn), heme oxygenase 1, and glutathione S-transferase A1, alongside immune mediators such as interleukin-8 (IL-8) and plasminogen activator inhibitor 1 (PAI-1). Syndecan-4, a key regulator of leukocyte trafficking and tissue remodeling, was also increased.

Proteins involved in amino acid metabolism and neurotransmitter pathways, including aromatic-L-amino-acid decarboxylase, kynurenine–oxoglutarate transaminase 1, and catechol O-methyltransferase, were differentially expressed, highlighting alterations in tryptophan and catecholamine metabolism. Dysregulation of energy metabolism was evident with increased expression of fructose-1,6-bisphosphatase 1, carbonic anhydrase 5A, NAD kinase, and pyridoxal phosphate homeostasis protein, indicating enhanced gluconeogenic flux and mitochondrial adaptation.

**Fig. 3:**
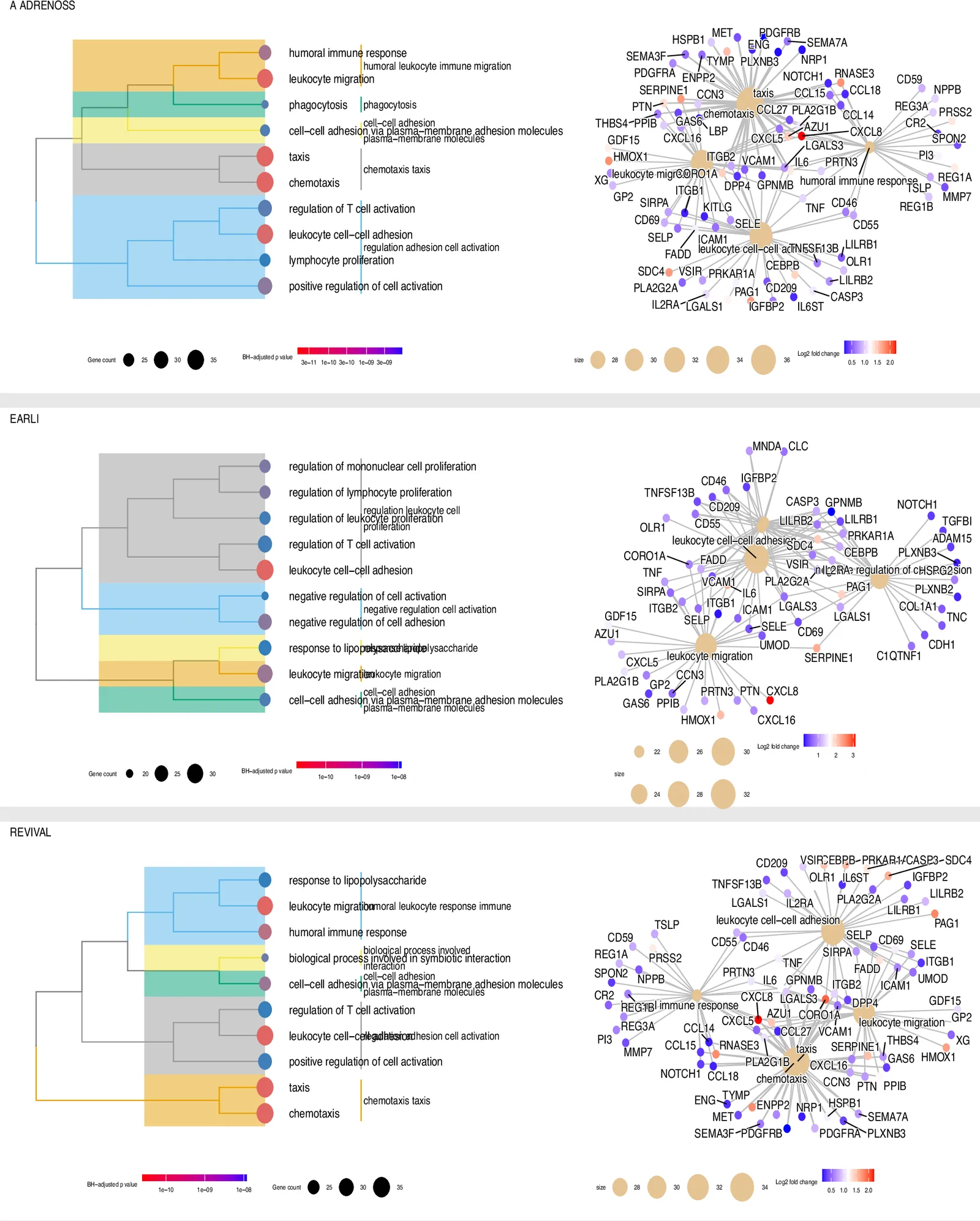
GO biological process enrichment of proteins upregulated in Subphenotype 1 across three cohorts. Proteins upregulated in Subphenotype 1 (higher-risk) relative to Subphenotype 2 (lower-risk) were identified using two-sided Wilcoxon rank-sum tests with Benjamini–Hochberg correction (BH-adjusted *p* < 0.05). In ADRENOSS, 315 proteins were upregulated in Subphenotype 1. In EARLI, 259 proteins were upregulated in Subphenotype 1. In REVIVAL, 309 proteins were upregulated in Subphenotype 1. Gene Ontology (GO) biological process enrichment was performed using over-representation analysis (clusterProfiler) with Benjamini–Hochberg correction; terms with FDR-adjusted *p* < 0.05 were considered significant. Over-representation analysis was one-sided, testing for enrichment only. Complete GO enrichment results are provided in the Source Data file. In the treeplots (left panels), each node represents an enriched GO term; node color indicates BH-adjusted *p*-value (red = most significant, blue = least significant) and node size indicates the number of associated genes. Related terms are grouped into clusters denoted by colored background bands. The top 10 non-redundant GO terms are shown per cohort. In the network plots (right panels), large beige nodes represent enriched GO terms and small colored nodes represent individual genes. Gene node color indicates Log₂ fold change (Subphenotype 1 minus Subphenotype 2), where red/orange indicates higher expression in Subphenotype 1 and blue indicates higher expression in Subphenotype 2. Node size reflects the number of associated genes per pathway. Edges connect genes to their associated pathways. The top 5 non-redundant GO terms are shown per cohort.

Over-representation analysis (ORA) confirmed distinct pathway enrichments between the two clusters. Subphenotype 1 demonstrated strong enrichment of immune activation, humoral immune responses, leukocyte migration, and extracellular matrix organization, driven by proteins consistently upregulated across all three cohorts (Supplementary Table 2). In contrast, Subphenotype 2 was enriched for pathways related to G-protein-coupled receptor signaling, wound healing and tissue repair, hemostasis, hypoxia response, and regulation of body fluid balance (Fig. 3 and Supplementary Fig. 4).

### Outcomes across subphenotypes

Despite similar baseline characteristics (Table 1), outcomes diverged markedly between subphenotypes across ADRENOSS, EARLI, and REVIVAL (Fig. 2—Part I, Supplementary Fig. 2—Part a, Table 2, Supplementary Table 3). Patients in Subphenotype 1 consistently exhibited higher mortality, greater risk of MAKE90 events, and fewer ICU-free days compared with those in Subphenotype 2. In survival analyses, the hazard ratios indicated a substantial risk gradient, with Subphenotype 1 associated with significantly poorer outcomes in all cohorts.

**Table 2:** Detailed outcomes across three cohorts

Outcomes	ADRENOSS						EARLI					REVIVAL				
	All	Sub. 1		Sub. 2	*P*-value^b^	*Q*-value^c^	All	Sub. 1	Sub. 2	*P*-value^b^	*Q*-value^c^	All	Sub. 1	Sub. 2	*P*-value^b^	*Q*-value^c^
	*N* = 319^a^	*N* = 132^a^		*N* = 187^a^			*N* = 169^a^	*N* = 59^a^	*N* = 110^a^			*N* = 611^a^	*N* = 275^a^	*N* = 336^a^		
28-Day mortality	94 (29%)	59 (45%)	35 (19%)		<0.001	<0.001	74 (44%)	40 (68%)	34 (31%)	<0.001	<0.001	172 (28%)	97 (35%)	75 (22%)	<0.001	0.006
90-Day mortality	122 (38%)	69 (52%)		53 (28%)	<0.001	<0.001	81 (48%)	44 (75%)	37 (34%)	<0.001	<0.001	210 (34%)	112 (41%)	98 (29%)	0.004	0.033
28-day ICU-Free Days	13.2 (10.8)	9.1 (10.6)		16.1 (9.9)	<0.001	<0.001	11.2 (11.2)	5.1 (8.8)	14.4 (11.0)	<0.001	<0.001	11.1 (10.5)	8.1 (9.5)	13.5 (10.6)	<0.001	<0.001
90-Day ICU-Free Days	49.8 (40.2)	36.7 (40.0)		59.0 (37.7)	<0.001	<0.001	42.3 (41.4)	19.1 (34.3)	54.5 (39.7)	<0.001	<0.001	44.9(38.6)	37.1 (37.4)	51.2(38.5)	<0.001	<0.001
MAKE within 90 days	209 (68%)	110 (85%)		99 (56%)	<0.001	<0.001	88 (52%)	47 (80%)	41 (37%)	<0.001	<0.001	377 (62%)	192 (70%)	185 (55%)	<0.001	0.003
Severe AKI within 7 days	81 (26%)	55 (42%)		26 (14%)	<0.001	<0.001	51 (30%)	32 (54%)	19 (17%)	<0.001	<0.001	229(37%)	145(53%)	84(25%)	<0.01	<0.01

*Sub* subphenotype, Severe AKI within 7 days was defined as an increase in serum creatinine to ≥2 times the baseline value or initiation of renal replacement therapy within 7 days following enrollment.

^a^Mean (SD); *n* (%).

^b^Two-sided Welch two-sample *t*-test for continuous variables; Fisher’s Exact Test when any expected cell count < 5, Pearson’s Chi-squared test otherwise, for categorical variables.

^c^*Q*-values were calculated using the Holm method to adjust for multiple comparisons.

### Parsimonious model and external validation

The biomarker selection pipeline (Supplementary Fig. 5) identified seven proteins as the optimal balance of parsimony and subphenotype prediction accuracy - Protein phosphatase inhibitor 2 (PPP1R2), C-type lectin domain family 1 member A (CLEC1A), Endoribonuclease LACTB2, Insulin-like growth factor-binding protein 7 (IGFBP7), Phosphoprotein associated with glycosphingolipid-enriched microdomains 1 (PAG1), Tissue inhibitor of metalloproteinases 1 (TIMP1), and CD97 antigen (adhesion G protein-coupled receptor E5)(supplementary Table 4). This clustering applied to this parsimonious set of seven proteins—Protein phosphatase inhibitor 2 (PPP1R2), C-type lectin domain family 1 member A (CLEC1A), Endoribonuclease LACTB2, Insulin-like growth factor-binding protein 7 (IGFBP7), Phosphoprotein associated with glycosphingolipid-enriched microdomains 1 (PAG1), Tissue inhibitor of metalloproteinases 1 (TIMP1), and CD97 antigen (adhesion G protein-coupled receptor E5)—reproduced the clustering patterns observed with the full proteomic model. In both ADRENOSS and EARLI, the seven-protein classifier maintained clear separation of the subphenotypes in PCA space, with PC1 explaining over 63% of the variance. Survival and outcome patterns mirrored those of the full model, confirming the stability and reproducibility of the reduced biomarker set (Fig. 2, Supplementary Figs. 2—Part c and 6).

**Fig. 4:**
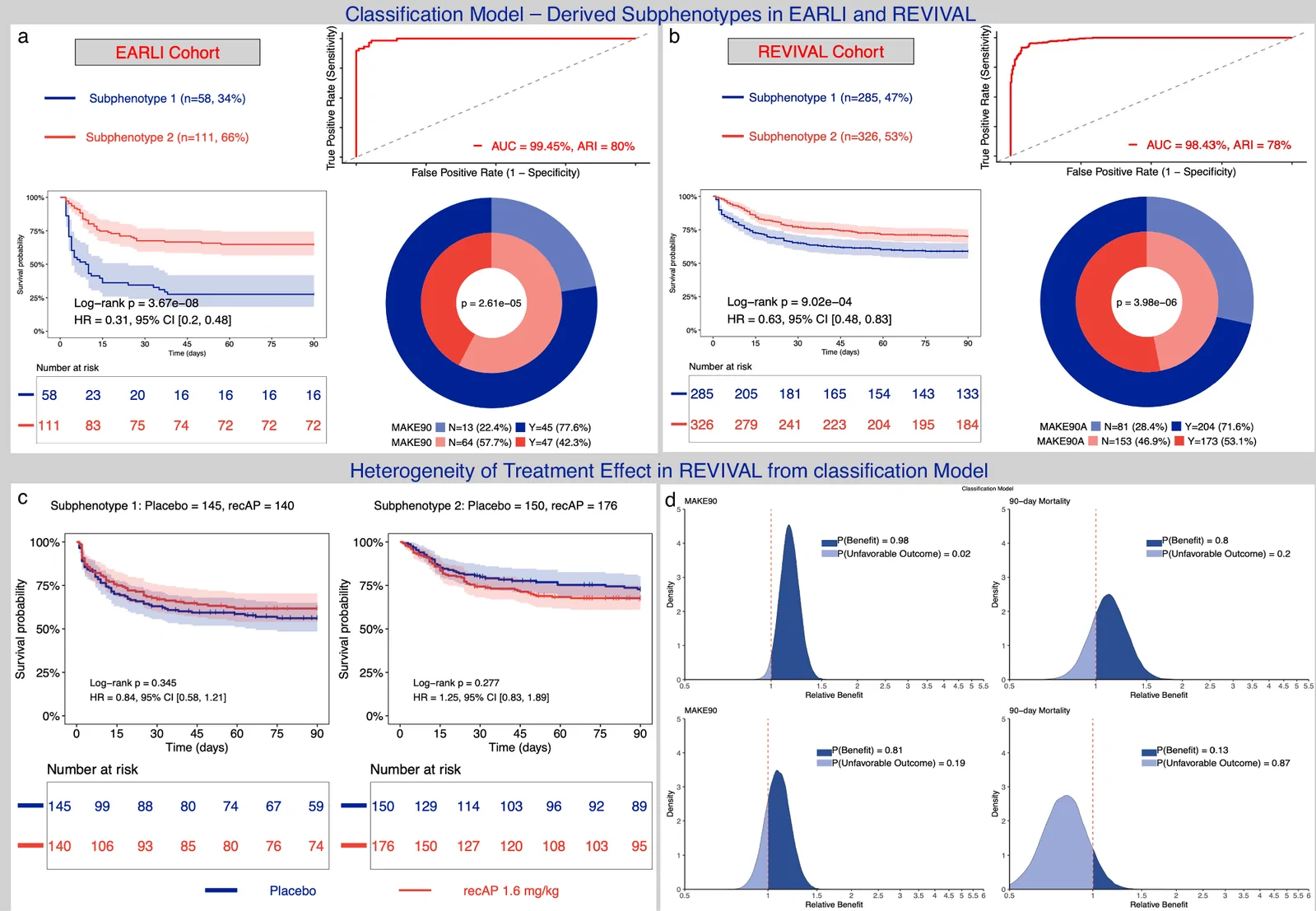
Validation of classification model-derived subphenotypes in EARLI and REVIVAL and heterogeneity of treatment effect in REVIVAL. Predicted versus clustered subphenotypes in EARLI (**a**) and REVIVAL (**b**) using a classification model trained on ADRENOSS with 7 proteins. ROC curves show discrimination (AUC, ARI); Kaplan–Meier curves show 90-day survival probability by subphenotype; doughnut charts show MAKE90 or MAKE90A distribution (N = no event, Y = event). Shaded bands represent 95% confidence intervals; numbers at risk are shown below each curve. HRs with 95% CIs were estimated using Cox proportional hazards regression; log-rank and *χ*² tests were two-sided; no adjustments were made for multiple comparisons. EARLI (a): Subphenotype 1 (*n* = 58, 34%), Subphenotype 2 (*n* = 111, 66%); AUC = 99.45%, ARI = 80%; log-rank *p* = 3.67 × 10^−8^, HR = 0.31 (95% CI [0.20, 0.48]); MAKE90 *p* = 2.61 × 10^−5^. REVIVAL (b): Subphenotype 1 (*n* = 285, 47%), Subphenotype 2 (*n* = 326, 53%); AUC = 98.43%, ARI = 78%; log-rank *p* = 9.02 × 10^−4^, HR = 0.63 (95% CI [0.48, 0.83]); MAKE90A *p* = 3.98 × 10^−6^. **c** Kaplan–Meier survival curves for recombinant alkaline phosphatase (recAP) versus placebo within each subphenotype in REVIVAL; shaded bands represent 95% confidence intervals; numbers at risk shown below each curve. Log-rank tests two-sided; no adjustments for multiple comparisons. Subphenotype 1 (placebo *n* = 145, recAP *n* = 140): log-rank *p* = 0.345, HR = 0.84 (95% CI [0.58, 1.21]). Subphenotype 2 (placebo *n* = 150, recAP *n* = 176): log-rank *p* = 0.277, HR = 1.25 (95% CI [0.83, 1.89]). **d** Posterior density distributions of the relative benefit of recAP versus placebo from non-informative Bayesian logistic regression. Dashed red line indicates RR = 1; dark blue = P(Benefit), RR ≥ 1; light blue = P(Unfavorable Outcome), RR < 1. Subphenotype 1 MAKE90: P(Benefit) = 0.98; 90-day mortality: P(Benefit) = 0.80. Subphenotype 2 MAKE90: P(Benefit) = 0.81; 90-day mortality: P(Benefit) = 0.13.

When applied to the EARLI and REVIVAL cohorts, the classifier achieved high discrimination with AUCs of 0.99 and 0.98 and ARIs of 0.80 and 0.78, respectively, demonstrating robust external validity. Predicted assignments aligned closely with clustering-derived labels, and survival and MAKE90 outcome patterns were consistent across cohorts (Fig. 4—Part a, b).

As an additional biological validation, we evaluated the parsimonious and full biomarker models in an independent healthy cohort, in which most samples clustered with the lower risk subphenotype (97.2% with the parsimonious model and 96.3% with the full model). Sensitivity analyses restricting sepsis-associated AKI diagnosis to within 48 h of admission showed similar biomarker rankings and subphenotype structure.

To evaluate whether demographic and clinical characteristics influenced subphenotype distribution, we included age, sex, and major comorbidities in both the clustering framework and in the subphenotype prediction models. Cluster assignments remained highly consistent with those derived from biomarkers alone (Jaccard index range, 0.80–0.88 across cohorts). Clinical variables demonstrated modest discrimination of biomarker-derived clustering assignments (AUC range, 0.64–0.79), and inclusion of clinical variables did not improve external prediction performance beyond biomarkers alone (Supplementary Table 5).

### Heterogeneity of treatment effect in REVIVAL

Among the 611 patients with biomarker data in the REVIVAL trial, 316 received ilofotase alfa, and 295 received placebo. Across subphenotypes, Subphenotype 1 was again associated with higher mortality and MAKE90 rates. Bayesian analyses incorporating non-informative priors demonstrated a high probability of benefit with ilofotase alfa in Subphenotype 1. In Subphenotype 2, the probability of benefit was lower, and posterior distributions suggested an increased probability of non-benefit. These findings, consistent in both full and parsimonious models, highlight potential heterogeneity of treatment response by subphenotype, with Subphenotype 1 showing a possible beneficial signal and Subphenotype 2 demonstrating attenuated or even unfavorable trends (Fig. 4—Part c, d and Supplementary Tables 6 and 7, Supplementary Figs. 7 and8).

## Discussion

This study applied an ensemble unsupervised clustering approach to high-dimensional proteomic data from patients with sepsis-associated acute kidney injury (SAKI) and consistently identified two biologically and prognostically distinct subphenotypes. These subphenotypes were reproducible across three large, independent cohorts—ADRENOSS, EARLI, and REVIVAL—highlighting the robustness, stability, and external validity of the classification strategy. By integrating ensemble methods that combine multiple clustering algorithms, this work strengthens the evidence that SAKI is a biologically heterogeneous condition and demonstrates how data-driven phenotyping can reveal meaningful subgroups in critically ill patients.

The two subphenotypes displayed consistent and biologically meaningful differences across cohorts. Subphenotype 1 was defined by a broad proteomic activation pattern, including heightened inflammatory, metabolic, and oxidative stress pathways, together with increased leukocyte trafficking, suggesting a maladaptive, hyperinflammatory host response marked by persistent metabolic reprogramming and redox imbalance. In contrast, Subphenotype 2 showed a comparatively muted profile, enriched for processes related to vascular adaptation and G-protein-coupled receptor signaling, consistent with a more regulated inflammatory state and preserved cellular homeostasis.

We also developed a parsimonious seven-protein model that accurately recapitulated the full 366-protein clustering structure. This reduced biomarker panel preserved the separation between subphenotypes, maintained high classification accuracy (AUCs of 0.99 and 0.98 in EARLI and REVIVAL, respectively), and replicated the prognostic and biological profiles observed with the full proteomic model. This 7-biomarker panel captured biologically and clinically meaningful subphenotypes of sepsis-associated AKI, supporting its potential use in both observational stratification and interventional trial enrichment. The inclusion of IGFBP7—a biomarker extensively studied in AKI and components of the FDA-cleared (associated with TIMP-2 in the NephroCheck test) lends biological plausibility to the model^10–12^. TIMP-1 was found to be associated with AKI^13–15^. These markers reflect early tubular stress and matrix remodeling, two key pathophysiologic processes in AKI. Their presence in our model not only affirms its relevance but also suggests the feasibility of bedside implementation using existing platforms. The addition of other proteins such as PPP1R2, CLEC1A, LACTB2, PAG1, and CD97 antigen offers both refinement in the classification and new avenues for mechanistic exploration and therapeutic targeting. The cell-of-origin analysis of the plasma proteins (Supplementary Fig. 9) suggests broad expression across the vasculature, immune cells, and parenchymal tissues, which aligns with the multiple organ involvement characteristic of sepsis as well as the remote organ injury described in AKI^16,17^. Interestingly, Fibroblast and connective tissue signatures recur across organs, which aligns with the role of fibroblasts as major producers of extracellular matrix and secreted signaling factors^18^. Endothelial representation also fits the known presence of endothelial-enriched proteins in plasma and the sensitivity of these proteins to systemic exposures^19^. No single cell type appears in all top tissues, which supports a mixed-origin model with shared stromal and vascular contributors plus tissue-restricted epithelial and immune sources. Of note, sepsis can shift both tissue programs and the circulating proteome, so the dominant sources in disease may differ from baseline patterns. This context matters for interpretation because RNA does not map cleanly to protein abundance across tissues, and secreted proteins can show discordance between the tissue where RNA is enriched and the site where protein accumulates or acts. The reproducibility of subphenotypes across diverse cohorts, collected in different geographic and clinical settings and analyzed on the same proteomic platform, supports the robustness and external validity of the findings. Practical steps will be needed to develop real-time biology measurements to test these candidate proteins in the setting of SAKI. Of note, the subphenotypes identified in our study were largely driven by biomarker patterns rather than demographic or clinical characteristics.

This biological divergence was associated with clinically meaningful differences in outcomes. Across all three cohorts, patients in Subphenotype 1 consistently exhibited higher mortality, increased incidence of MAKE90 events, and fewer ICU-free days, despite broadly similar baseline demographic and clinical characteristics. The clear prognostic gradient underscores the potential of proteomic endotyping to capture risk not apparent from traditional clinical parameters, thereby offering a more refined approach to risk stratification in SAKI. Exploratory analyses in the REVIVAL cohort revealed signals of heterogeneity of treatment effect (HTE) with ilofotase alfa. In Subphenotype 1, Bayesian analyses indicated a high posterior probability of clinical benefit with minimal probability of non-benefit. Conversely, patients in Subphenotype 2 demonstrated attenuated probabilities of benefit and higher probabilities of non-benefit. The consistency of the signal across the full and parsimonious models highlights the potential for biologically-informed enrichment strategies in future interventional studies. The lower proportion of patients with subphenotype 1 in EARLI may be related to earlier enrollment in the course of the disease, as EARLI enrolled patients from the emergency department (versus the ICU). Prospective validation of subphenotype-specific responses is warranted to test whether biomarker-guided enrichment can improve the efficiency and success of clinical trials in SAKI.

Our findings align with, but also extend, prior work on subphenotyping in critical illnesses. For example, Bhatraju and colleagues, using latent class analysis (LCA) of 29 clinical and biomarker variables, identified two reproducible AKI subphenotypes (AKI-SP1 and AKI-SP2) across independent cohorts (but not restricted to sepsis)^18^. AKI-SP2 was associated with greater illness severity, shock, and respiratory failure, along with higher systemic inflammation and endothelial injury markers such as IL-8, soluble TNF receptor-1, and Angiopoietin-2^20^. In the present study, we evaluated overlap with these previously reported biomarkers and found that Ang1/Ang2 and sTNFR1 were only moderately associated with our biomarker-derived subphenotypes, suggesting that our finding subphenotypes are not primarily driven by these markers. Logistic prediction models based on Ang1/Ang2 and sTNFR1 achieved only modest AUCs of 0.669 for the full model clusters and 0.655 for the parsimonious clusters. Similarly, Calfee and colleagues have described hyperinflammatory and hypoinflammatory subphenotypes in ARDS and sepsis with prognostic and therapeutic implications. Our Subphenotype 1 appears to share key features with these hyperinflammatory, high-risk phenotypes, including heightened inflammatory signaling, oxidative stress, and metabolic activation, while Subphenotype 2 shares characteristics of more adaptive or hypoinflammatory states^11^. However, our study focuses specifically on SAKI, and the use of high-dimensional proteomics in our analysis allows for a more granular characterization of molecular pathways, particularly around metabolic reprogramming and redox balance, that were less evident in prior studies using targeted biomarker panels. These distinctions suggest that while the biological axes of inflammation and vascular dysfunction are shared across syndromes, SAKI-specific proteomic endotyping may reveal unique therapeutic targets.

This study has several strengths. First, it focuses exclusively on sepsis-associated AKI, the most common and clinically relevant AKI phenotype in critical illness, providing a more homogeneous baseline population than prior studies. Second, it leverages a comprehensive proteomic platform, enabling high-resolution biological profiling across multiple independent, prospectively collected cohorts. Third, the use of ensemble clustering methods minimizes algorithm-specific biases and enhances the stability of the clustering solution. Fourth, the reproducibility of findings across diverse cohorts and care settings supports their generalizability. Finally, by integrating pathway-level with cell-of-origin analyses and developing a clinically applicable seven-protein model, the study bridges mechanistic insights and clinical translation, moving the field closer to actionable precision medicine strategies in critical care nephrology.

Several limitations merit consideration. The observational nature of the discovery and validation cohorts precludes causal inference between subphenotype membership and outcomes. Although validation was performed in two independent cohorts, all analyses relied on the same proteomic platform, and generalizability to other platforms or populations requires further study. The model was developed using features from primary sepsis data. While this approach anchors our model to clinical sepsis, it is important to note that heterogeneity in regard to hospital assay platforms, patient comorbidities, sepsis presentation, and sample handling can all impact the fidelity of the model in predicting SAKI at the level of individual patients. During model development, these factors were controlled in part by using an ensemble approach, which reduces sensitivity to stochasticity. Additionally, differences across cohorts were further examined through pooled ensemble clustering across ADRENOSS, EARLI, and REVIVAL (*n* = 1099). In the pooled analysis, the two-subphenotype structure was supported by the lowest proportion of ambiguous clustering (PAC), indicating stability of the clustering structure across cohorts. Remaining variance arising from clinical heterogeneity could be mitigated by ensuring a standardized normalization approach to all input variables and the utilization of anomaly prediction models to discount data whose variance would translate into low confidence predictions. As more training data becomes available, periodic model retraining after deployment would also contribute to a reduction in variance. The exploratory treatment effect analyses in REVIVAL were post hoc and underpowered for a frequentist analysis, limiting definitive conclusions about differential drug response. They should be considered exploratory. In addition, while the seven-protein parsimonious model enhances feasibility, it requires further prospective testing in real-world settings to confirm accuracy and operational performance. Notably, the 7-feature parsimonious model relies on biomarkers that are measurable but not routinely available in most institutions (IGFBP-7 is commercially available and FDA-cleared). A key barrier to its clinical usability is therefore the capacity of a laboratory to assay these biomarkers. The utility of this model could potentially be enhanced by the development of a combined assay capable of measuring all 7 biomarkers simultaneously.

To conclude, this study demonstrates that patients with SAKI can be reliably classified into biologically and prognostically distinct subphenotypes using ensemble proteomic clustering, with consistent replication across independent cohorts and validation using a simplified biomarker panel. These findings highlight the potential of proteomic endotyping to refine prognostication, deepen understanding of SAKI pathophysiology, and enable precision therapeutic approaches. Future research should focus on real-time implementation of the parsimonious model and prospective trials designed to test targeted therapies in biomarker-defined subgroups.

## Methods

### Study population

The current research was qualified as Exempt by UCSF IRB (IRB #: 26-46890). We used data from three independent prospective studies for this analysis. The ADRENOSS-1 study served as the discovery cohort. The ADRENOSS study (ClinicalTrials.gov Identifier: NCT02393781) was a prospective observational multinational study that included patients admitted to the ICU with sepsis or septic shock using the sepsis-3 definition^21^. The EARLI cohort served as a validation cohort. The EARLI study is a prospective observational study of patients admitted to the ICU via the Emergency Department at two University of California, San Francisco-affiliated hospitals^22,23^. The REVIVAL trial served as a second validation cohort to explore the potential heterogeneity of treatment effect across subphenotypes^24^ (NCT04411472). The REVIVAL trial was a multinational randomized trial investigating the impact of ilofotase alfa on outcomes in patients with SAKI^24^. All three studies were previously published and conducted with appropriate ethical approvals by relevant institutional ethics committees across participating European sites. EARLI was approved by the UCSF Institutional Review Board #10-02852). Full details of ethical approvals are provided in the original study publications. This study complied with all relevant ethical regulations. The present secondary analysis of EARLI was covered under UCSF Institutional Review Board approval #10-02852. For ADRENOSS-1 and REVIVAL, the original ethics committee approvals and participant informed consent authorized secondary analyses of de-identified biomarker and outcome data; accordingly.

### Definition of early sepsis-associated acute kidney injury

SAKI was defined as a rise of serum creatinine (Screat) value of 0.3 mg/L or more than 50% higher than the baseline value within 7 days from admission^25^. In REVIVAL, pre-AKI creatinine values were used as the baseline value. In EARLI, baseline creatinine was defined as the most recent serum creatinine value measured within 6 months of admission, when available (66 of 169 patients). First, the value upon admission was used otherwise. In ADRENOSS, the baseline value was assessed using the SCreat calculated back from the Modification of Diet in Renal Disease Study (MDRD) equation set to 75 ml/min per 1.73 m^2^ ^26^.

### Outcomes

The primary outcome of the study was 90-day all-cause mortality. Secondary outcomes were 28-day mortality, ICU-free days at day 28 and day 90, and major adverse kidney events (MAKE)^1^, defined as death or need for Renal Replacement Therapy (RRT) within 90 days or absence of renal recovery at day 90, defined as an estimated glomerular filtration rate (eGFR) rate at least 25% lower than the baseline value.

### Biomarkers measurements

In all 3 cohorts, blood was collected at study enrollment. The labeled EDTA aliquots were stored at −80 °C. Plasma proteomic profiling was performed using the Olink proximity extension assay, an oligonucleotide-labeled antibody assay for high-specificity, high-dimensional multiplexing that allows measurement of low-abundance (pico-gram (pg) range) proteins. For each protein, a pair of oligonucleotide-labeled antibody probes binds to the targeted protein, and if the two probes are in close proximity, a PCR target sequence is formed by a proximity-dependent DNA polymerization event. The resulting sequence is subsequently quantified by real-time PCR using the Fluidigm BioMark™ HD real-time PCR platform. We used a panel of 366 biomarkers (i.e., cardiometabolic panel) representing a selection of biomarkers exploring systemic inflammatory and immune responses as well as cardiovascular injury. Each assay includes calibration curves using recombinant antigens, with data presented as Normalized Protein eXpression (NPX) values plotted against protein concentration (in pg/mL). Protein expression values were obtained as NPX values provided by the Olink platform. NPX values are reported on a log_2_ scale and normalized using Olink’s standardized preprocessing pipeline; negative values may occur because of this normalization.

### Ensemble clustering

Subphenotype identification was conducted using unsupervised machine learning methods applied to the proteomic datasets. Clustering is a key technique for identifying data-driven subgroups, but results can vary depending on the algorithm, parameter initialization, sensitivity to outliers, and tree-cutting thresholds in hierarchical methods. To reduce this variability, we used ensemble clustering (Fig. 1—Part I), which integrates multiple algorithms to generate consensus clusters. This approach improves stability, minimizes biases specific to individual methods, and enhances robustness to noise. The methodology enables robust identification of groupings within data that are less sensitive to the variability that can occur when individual unsupervised models are used for cluster identification^16^. Ensemble clustering is increasingly utilized for its ability to produce stable, data-driven classifications, with applications across genomics, proteomics, cancer subtyping, and disease taxonomy, though it remains underutilized in sepsis research.

Because NPX values are already log-transformed and normalized by the Olink platform, no additional feature scaling or normalization was applied prior to clustering, and the same preprocessing approach was used consistently across all three cohorts.

### Candidate clustering algorithms

The ensemble incorporated 12 algorithms spanning diverse methodologies, including model-based, density-based, partitioning, hierarchical, neural-network, and biclustering methods. These included *K*-modes for categorical clustering, PAM for robustness to outliers, Fuzzy *C*-means for soft clustering, and Gaussian mixture modeling (GMM) for overlapping clusters. HDBSCAN accounted for variable density and noise detection, while spectral clustering and affinity propagation allowed detection of complex structures without predefining cluster numbers. Hierarchical clustering (HC), DIANA, self-organizing maps (SOM), non-negative matrix factorization (NMF), and latent block modeling captured hierarchical, neural network-based, and biclustering perspectives. Specifically, *K*-modes adapts *K*-means for categorical variables by using modes instead of means to define cluster centers; PAM selects representative data points (medoids) rather than centroids, offering increased robustness to outliers; Fuzzy *C*-Means enables partial membership in multiple clusters; GMM models data as originating from multiple Gaussian distributions, accommodating overlapping and elliptical clusters; HDBSCAN builds on DBSCAN to allow variable-density clusters while identifying noise as outliers; Spectral Clustering identifies complex, non-convex clusters via the eigenstructure of a similarity matrix; and Affinity Propagation clusters data through iterative message passing without requiring pre-specification of cluster numbers. This diverse algorithm portfolio ensured that the ensemble captured robust, reproducible structures, reducing dependency on any single method’s assumptions.

Cluster stability was assessed using the proportion of ambiguous clustering (PAC) metric across solutions with two to six clusters. A two-cluster solution demonstrated the lowest PAC, indicating the highest stability, and this finding was corroborated by internal validation with the elbow method, silhouette analysis, and gap statistic. All three methods suggested that two clusters best fit our data. Ensemble clustering was implemented in the diceR package (v3.1.0) in R v4.4.2^27^.

### Consensus clustering and validation

Cluster outputs from the individual algorithms were evaluated using rank aggregation guided by multiple internal validity metrics. These included the Dunn index (assessing compactness and separation), the *C* index (quantifying intra- and inter-cluster distances), the Baker–Hubert gamma statistic (correlating pairwise distances with clustering), and silhouette width (evaluating cohesion and separation at the individual data point level). The complete set of clustering results from the twelve algorithms was used to derive the consensus clustering assignments via latent class analysis (LCA), a probabilistic model that estimates membership probabilities to identify latent subgroups (Fig. 1, Panel I). The resulting LCA class predictions were used as the final consensus cluster assignments. Principal component analysis (PCA) was used for visualization, reducing the dimensionality of the data to illustrate separation between the two clusters and quantifying the proportion of variance explained (Fig. 1—Part II). PCA also quantifies the proportion of variance captured by the principal components, providing insight into how well the clusters explain the overall data variability.

### Differential expression and pathway analysis

To characterize biological differences between clusters, two-sided Mann–Whitney *U* tests were applied to NPX protein values using the Olink® Wilcox function. Specifically, the Olink Wilcox function from the Olink® Analyze R package was used to perform two-sided tests on NPX values for each protein across the two clusters. To account for multiple comparisons, *p*-values were adjusted using the Benjamini–Hochberg procedure to control false discovery rate (FDR). Proteins with FDR-adjusted *p*-value (*q*-value) < 0.05 were considered significantly differentially expressed and were used as input for functional enrichment analyses.

*P*-values were adjusted with the Benjamini–Hochberg procedure, and proteins with FDR-adjusted *p*-values below 0.05 were considered significant.

Significant proteins were then analyzed for pathway enrichment using the clusterProfiler R package (4.16.0)^28^. Proteins with adjusted *p*-values ≤ 0.05 were mapped to Entrez Gene IDs, and over-representation analysis (ORA) via enrichGO and enrichKEGG identified enriched Gene Ontology domains, including biological process, molecular function, and cellular component, as well as canonical KEGG pathways. Pathway-level multiple testing correction was applied using the Benjamini–Hochberg procedure. Terms were mapped to human annotations using org.Hs.eg.db, and significant terms were reported after FDR correction for interpretability. Enriched terms were converted to gene symbols via the setReadable function. Analyses were conducted using R version 4.4.2.

### Parsimonious biomarker clustering

While clustering based on the complete protein panels (366 proteins in ADRENOSS and EARLI; 359 in REVIVAL) identified well-separated subphenotypes, we sought to determine whether comparable stratification could be achieved with fewer proteins to enhance clinical applicability and interpretability. To this aim, we implemented a three-stage biomarker selection pipeline to identify the most discriminative proteins while minimizing redundancy and measurement costs (see the “Methods” section and Supplementary Fig. 5). We also examined gene expression across normal human tissues using the CELLxGENE database^29^ to define the tissue context of the parsimonious-protein model.

The pipeline consisted of three stages. In the first stage, we identify the stable protein list by applying four machine learning algorithms (Random Forest, LASSO, XGBoost, and Nearest Shrunken Centroid) with repeated cross-validation in each cohort. Proteins selected in 80% or more of all repetitions in each cohort were considered stable. The final list is the intersection of stable proteins across the three cohorts. In Stages 2 and 3, ADRENOSS was used as the discovery cohort. In Stage 2, the stable protein list obtained from Stage 1 was ranked by averaging their normalized importance scores across the four algorithms (RF, LASSO, XGBoost, NSC). In Stage 3, the parsimonious protein set was identified using cross-validation. The procedure consisted of two logistic regression loops: in the inner loop, the optimal number of top-ranked proteins was selected based on the smallest Bayesian information criterion (BIC) across the four testing folds; in the outer loop, the model using the chosen *k* was evaluated on the held-out test fold. The procedure resulted in a 7-protein model.

### External validation

We evaluated consistency of the cluster-identified subphenotypes across the three cohorts using a Super Learner ensemble classifier trained on the parsimonious protein set in ADRENOSS and tested in EARLI and REVIVAL^30^. To make the biomarker panels across cohorts comparable, protein measurements (Olink NPX format) were batch-corrected using the ComBat method (Johnson et al. 2007)^31^ implemented in the sva R package prior to training and testing the Super Learner ensemble, which combined three base learners: Random Forest, XGBoost, and elastic net logistic regression. Model performance was evaluated using the area under the ROC curve (AUC) and adjusted Rand index (ARI) (Supplementary Fig. 10).

To evaluate biological plausibility, the classification model was also applied to an independent cohort of healthy control samples (*n* = 108).

### Heterogeneity of treatment effect in REVIVAL

To test whether subphenotypes predicted differential response to ilofotase alfa at 1.6 mg/kg in the REVIVAL cohort, Bayesian analyses were performed using non-informative priors to ensure data-driven estimates^31,32^. Two complementary methods were applied: (1) Bayesian regression to estimate the probability of adverse outcomes, including 90-day mortality, and MAKE90, within each subphenotype and treatment arm; and (2) a Bayesian A/B test to generate posterior risk distributions comparing the treatment and placebo groups. To test whether the treatment effect differed between subphenotypes, a Bayesian logistic regression model was fit for the full dataset with an interaction term between treatment arm and subphenotype. The interaction odds ratios (ORs) quantified the differential treatment effect between subphenotypes. Because the outcomes (90-day death, MAKE90) are adverse events, an interaction OR < 1 indicates greater treatment benefit in subphenotype 1 compared to subphenotype 2, while OR > 1 indicates lesser treatment benefit.

Bayesian regression estimated the probability of adverse outcomes,including 90-day and 28-day mortality, and MAKE90, within each subphenotype and treatment arm. A Bayesian A/B test was also conducted to derive posterior risk distributions comparing treatment and placebo groups. We additionally used a Bayesian logistic regression model, with non-informative priors, including an interaction term between subphenotype and treatment arms. The interaction odds ratios quantified whether the treatment effect differed between subphenotypes. An interaction OR < 1 indicates greater treatment benefits, while OR > 1 indicates lesser treatment benefits. We also explored the treatment effect within each cluster using separate Cox models.

### Tissue-level expression analysis of candidate plasma proteins

To evaluate the tissue sources of the seven candidate plasma proteins, we analyzed gene expression across normal human tissues using publicly available CELLxGENE expression summaries (PMID: 39607691). We focused on the corresponding human gene symbols: PPP1R2, CLEC1A, LACTB2, IGFBP7, PAG1, TIMP1, and ADGRE5.

To restrict the analysis to generalizable organ systems, we excluded reproductive, developmental, pregnancy-related, and fluid-derived annotations. Specifically, we removed ovary, testis, uterus, fallopian tube, prostate gland, placenta, embryo, yolk sac, milk, pleural fluid, cerebrospinal fluid, saliva, and similar non-organ labels. We also excluded anatomical regions and microanatomical structures that do not represent whole organs, including the abdomen, axilla, head, neck, forelimb, hindlimb, lamina propria, mucosa, paracolic gutter, and tendon of semitendinosus.

For each tissue, we computed a composite signature score defined as the mean scaled expression (*z*-score) across the seven genes. We ranked tissues by this composite score to identify those with the highest enrichment of the candidate gene set. We also identified, for each gene individually, the tissue with the highest raw expression value. Scaled expression heatmaps were generated to visualize the distribution of each gene across tissues.

### Cell type-level analysis within top tissues

To identify candidate cellular sources, we next examined cell type-level expression within the five highest-scoring tissues: pleura, esophagus, vasculature, gallbladder, and musculature. We imported the corresponding CELLxGENE subcategory expression tables into R and harmonized column names as described above.

We excluded entries labeled as “aggregated,” “cell,” or “cells,” as these represent non-specific annotations. For each remaining cell type within each tissue, we calculated the same composite signature score as the mean scaled expression across the seven genes. We also computed the percentage of cells expressing each gene using the reported number of expressing cells divided by the total cell count. Within each tissue, we ranked cell types by composite signature score and retained the top 20 cell types for downstream analysis. We visualized gene-level expression patterns using dot plots, with dot color representing scaled expression and dot size representing the percentage of cells expressing the gene.

### Overlap analysis of cell types across tissues

To assess whether the candidate proteins arise from shared or tissue-specific cellular sources, we evaluated the overlap of the top 20 cell types across the five highest-scoring tissues. For each tissue, we defined a set consisting of its top 20-ranked cell types. We then calculated, for each cell type, the number of tissues in which it appeared among the top 20.

We summarized overlap patterns by counting the number of cell types present in one, two, three, four, or five tissues. We visualized these overlaps using an UpSet plot to display the size of each tissue combination and the number of shared cell types per intersection. This approach allowed us to distinguish tissue-specific cell types from those shared across multiple organs.

All analyses were performed in R. Plots were generated using ggplot2 and ComplexUpset. (https://ggplot2.tidyverse.org) No additional statistical testing was applied, as this analysis was descriptive and intended to characterize tissue and cellular expression patterns of the candidate gene set.

### Sensitivity and ancillary analyses

To assess the robustness of our findings, we conducted a series of sensitivity and ancillary analyses. Subphenotype assignments were stable under healthy control validation, restriction to early-onset AKI (within 48 h), incorporation of clinical variables, and variation in sample size.

#### Evaluation using a healthy control sample

To evaluate the biological plausibility of the identified subphenotypes, we examined model behavior in an independent cohort of healthy control samples (*n* = 108), with plasma biomarkers measured using the Olink platform. Biomarker values of the healthy control samples were standardized using the mean and standard deviation from the ADRENOSS cohort to maintain consistency with the model training framework. First, classification models trained on ADRENOSS were applied to predict subphenotype assignments in healthy control samples. Next, we also performed unsupervised clustering using the same ensemble clustering procedure applied in the primary analysis on the combined ADRENOSS and healthy samples (*n* = 427). Results are reported in Supplementary Table 8.

#### Early (within 48 h) sepsis-associated acute kidney injury (SAKI)

To evaluate whether the timing of sepsis-associated AKI influenced subphenotype identification, sensitivity analyses were restricted to AKI cases occurring within 48 h of admission. In ADRENOSS and EARLI, we identified patients meeting AKI criteria within 48 h of admission (ADRENOSS: *n* = 216; EARLI: *n* = 76). In the REVIVAL study, AKI within 48 h of admission was an inclusion criterion; therefore, all participants met both the 48-h and 7-day AKI definitions. For ADRENOSS and EARLI, we repeated clustering analysis using the same ensemble clustering pipeline and modeling framework as in the primary analysis, restricting the cohort to patients with AKI within 48 h. Biomarker importance rankings were derived using the same approach applied in the primary analysis. Rank agreement was calculated using Spearman correlation, with higher correlation indicating greater consistency in biomarker importance across AKI definitions. Spearman correlation tests were two-sided with no adjustments for multiple comparisons.

### Comparison with angiopoietin/TNFR subphenotypes

To assess overlap with previously reported sepsis-associated AKI subphenotypes, we examined angiopoietin-1 (Ang1), angiopoietin-2 (Ang2), and soluble TNF receptor-1 (sTNFR1), which were included in prior subphenotyping work by Bhatraju et al. In the EARLI cohort, all three biomarkers were available for 108 of 169 patients. Following the analytic strategy described by Bhatraju et al., we fit logistic regression models using Ang2/Ang1 and sTNFR1 as predictors, with clustering assignments from either the full biomarker model or the parsimonious biomarker model as the outcome. Model performance was assessed using the area under the receiver operating characteristic curve (AUC). We also performed unsupervised clustering using Ang1, Ang2, and sTNFR1 and compared these clusters with those derived from the parsimonious biomarker panel using the Jaccard index and the adjusted Rand index.

### Incorporation of clinical variables

To examine whether differences in subphenotype proportions across cohorts reflected variation in demographic characteristics, comorbid conditions, or markers of acute illness severity, we incorporated age, sex, hypertension, chronic obstructive pulmonary disease, diabetes, coronary artery disease, congestive heart failure, chronic liver disease, chronic kidney disease, vasopressor use, and mechanical ventilation into the clustering framework. We repeated the clustering framework within each cohort after inclusion of clinical variables, and agreement with biomarker-only clustering assignments was calculated using the Jaccard index. We also evaluated whether clinical variables alone could predict biomarker-derived clustering membership using a logistic regression model, and whether including clinical variables improved external prediction performance using the same classification framework as in the primary analysis

#### Cluster stability and sample size considerations

To evaluate cluster stability and potential sample size effects, we assessed stability using a resampling-based consensus clustering framework (diceR). For each cohort separately, we evaluated cluster solutions ranging from *k* = 2 to *k* = 6 and quantified stability using the proportion of ambiguous clustering (PAC), where lower values indicate greater partition stability. To further examine whether cluster number was sensitive to sample size, we repeated the consensus clustering procedure using pooled data from ADRENOSS (*n* = 319), EARLI (*n* = 169), and REVIVAL (*n* = 611), yielding a combined dataset of 1099 patients.

#### Frequentist evaluation of treatment effects

To assess whether results were dependent on the Bayesian framework, we performed frequentist Cox proportional hazards analyses within each subphenotype. A model including a treatment-by-subphenotype interaction term was also fit. All Cox proportional hazards analyses were two-sided and unadjusted.

### Statistical analysis

All statistical analyses were performed in R (version 4.5.1). The following R packages were used: diceR (v3.1.0) for consensus clustering; tidyLPA (v2.0.2) for latent class analysis; OlinkAnalyze (v4.5.0) for Olink proteomics analysis; sva (v3.58.0) for ComBat batch effect correction; survival (v3.8.6) and survminer (v0.5.2) for survival analysis; survRM2 (v1.0.4) for restricted mean survival time analysis; coin (v1.4.3) for permutation-based log-rank testing; clusterProfiler (v4.18.4) and enrichplot (v1.30.5) for gene ontology enrichment analysis; org.Hs.eg.db (v3.22.0) and GO.db (v3.22.0) for gene annotation; biomaRt (v2.66.1) for gene identifier mapping; SuperLearner (v2.0.40) and caret (v7.0.1) for classification modelling; pROC (v1.19.0.1) for receiver operating characteristic analysis; gtsummary (v2.5.0) for summary tables; clValid (v0.7) and fpc (v2.2.14) for clustering validation; cluster (v2.1.8.2) for silhouette analysis; factoextra (v2.0.0) for clustering visualization; umap (v0.2.10.0) for dimensionality reduction; lmtest (v0.9.40) for likelihood ratio testing; VennDiagram (v1.8.2) for Venn diagram visualization; brms (v2.23.0) for Bayesian logistic regression modelling; bayesAB (v1.1.0) for Bayesian A/B testing; bayesplot (v1.15.0) for Bayesian model diagnostics; bayestestR (v0.17.0) for Bayesian statistical inference.

## Supplementary information

Supplementary Information

Transparent Peer Review file

## Source data

Source data

## Data Availability

The individual patient-level clinical and outcome data from ADRENOSS-1 (Mebazaa et al., ref. ^15^), EARLI (Sinha et al., refs. ^11^^,^^12^), and REVIVAL (Pickkers et al., ref. ^13^) are available under restricted access because of patient-privacy requirements and the ethical and legal terms of the original studies’ informed consent and data use agreements. Access can be obtained by contacting the corresponding author (M. Legrand; Matthieu.legrand@ucsf.edu) and the data custodians of the source studies. Requestors can expect an initial response within 2 weeks of contact. Access requires a signed data use agreement restricting use to the analyses specified in the approved protocol, prohibiting redistribution to third parties, and prohibiting any attempt at participant re-identification. The plasma Olink proteomics data generated in this study have been deposited in the PRIDE Archive Affinity Proteomics resource (PRIDE-AP) under accession number PAD000052. The source data underlying all figures in this manuscript are provided as a supplementary CSV file. The gene expression data used in this study are available in the CZ CELLxGENE Discover database (CZI Cell Science Program et al., ref. ^18^) under https://cellxgene.cziscience.com/. Source data are provided with this paper.

## Code availability

Clustering analysis code used in this study is available and permanently archived via Zenodo (https://doi.org/10.5281/zenodo.20647967) and Code Ocean (https://doi.org/10.24433/CO.5394379.v1. The repository is cited in the reference list.
