# Supplementary material for: Plasma proteomics defines two reproducible subphenotypes of sepsis-associated acute kidney injury with distinct outcomes
Source: Nat Commun. 2026 Aug 19;17:10575. doi: 10.1038/s41467-026-76889-3 (PMC13646287; doi:10.1038/s41467-026-76889-3)
Supplement: Supplementary file 1 [file 41467_2026_76889_MOESM1_ESM.pdf]

Plasma proteomics defines two reproducible subphenotypes of sepsis-associated acute kidney injury with distinct outcomes

Supplementary Information

Table of Contents

Supplementary Figures

Supplementary Figure 1: Flow diagram of EARLI cohort selection.

Supplementary Figure 2: Comparison of Subphenotypes from the full biomarker model (a–b) and the parsimonious 7-protein model (c–d) in REVIVAL.

Supplementary Figure 3: Correlation of shared top 10% clustering driving proteins across cohorts.

Supplementary Figure 4: Gene Ontology (GO) Biological Process enrichment of proteins upregulated in Subphenotype 2 across three cohorts: ADRENOSS (a), EARLI (b), REVIVAL (c).

Supplementary Figure 5: Parsimonious model pipeline.

Supplementary Figure 6: Comparison of upregulated (a) and downregulated (b) proteins between the full and parsimonious models in ADRENOSS, EARLI, and REVIVAL.

Supplementary Figure 7: Bayesian A/B test results for treatment effects on MAKE90 and 90-day mortality (DEAD90) in the REVIVAL cohort.

Supplementary Figure 8: Bayesian A/B test results for treatment effects within Subphenotypes in the REVIVAL parsimonious model.

Supplementary Figure 9: Cell-of-origin of the plasma proteins.

Supplementary Figure 10: Validation of ADRENOSS - classification model pipeline.

Supplementary Tables

Supplementary Table 1: Tables of shared top 10% proteins from the cluster driving partition.

Supplementary Table 2: Cross-Cohort Shared Top 10% Upregulated Proteins in Subphenotype 1.

Supplementary Table 3: Detailed major adverse kidney events at 90 days (MAKE90) components across three cohorts.

Supplementary Table 4: List of the 7 proteins used in the parsimonious model.

Supplementary Table 5: Clinical Outcomes by Cluster Across Three Cohorts: Parsimonious + Clinical vs. Parsimonious Models.

Supplementary Table 6: Detailed treatment effect by cluster of recombinant alkaline phosphatase (recAP, ilofotase alfa).

Supplementary Table 7: Bayesian Logistic Regression for heterogeneity of treatment effect (HTE) with interaction terms in REVIVAL.

Supplementary Table 8. Outcome associations by subphenotype in ADRENOSS patients after inclusion of healthy control samples in the clustering framework (n=319 patients; n=108 healthy controls).

Supplement Results: Sensitivity and Ancillary Analyses

Supplementary Figures

Supplementary Figure 1. Flow diagram of EARLI cohort selection.

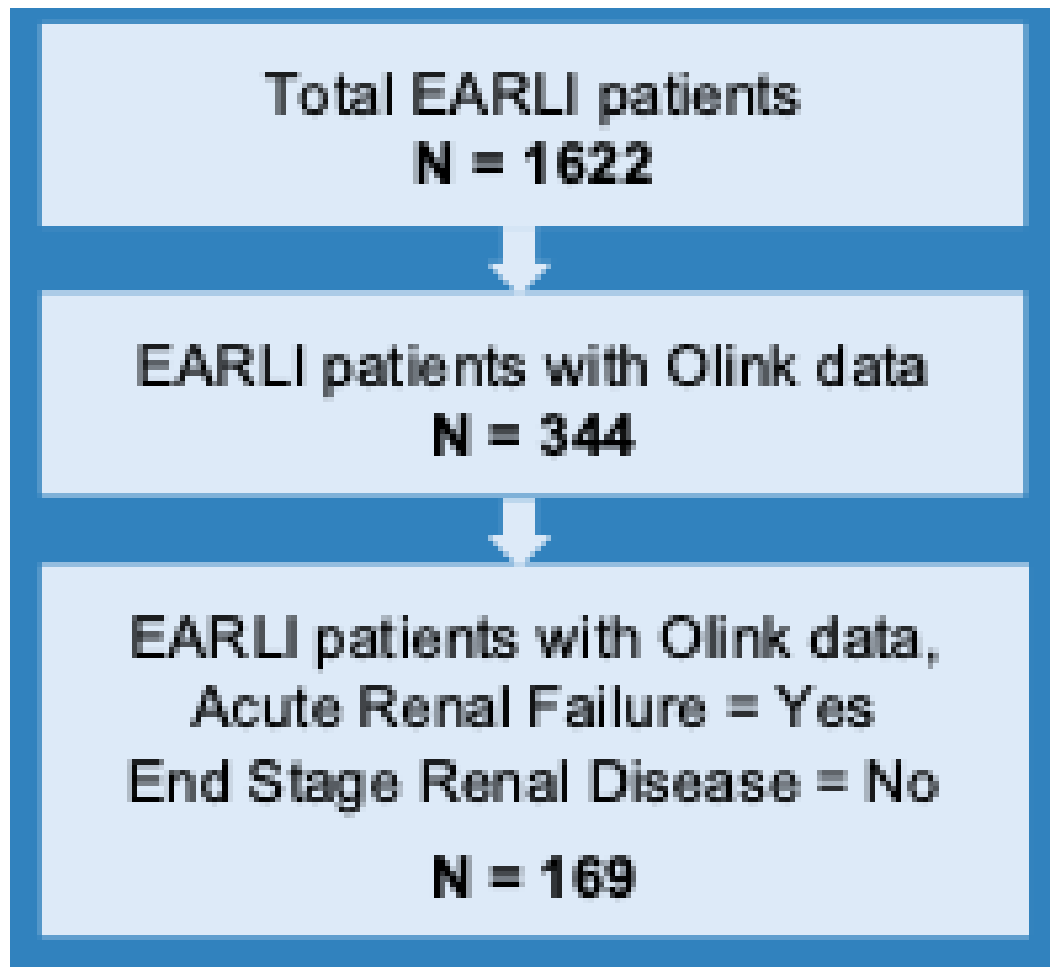

Flow diagram showing the sequential selection of patients from the EARLI cohort for inclusion in the study. Of 1,622 total EARLI patients, 344 had available Olink proteomics data. After further exclusion of patients without acute renal failure (ARF) or with end-stage renal disease (ESRD), 169 patients were included in the final analysis.

Supplementary Figure 2. Comparison of Subphenotypes from the full biomarker model (a–b) and the parsimonious 7-protein model (c–d) in REVIVAL.

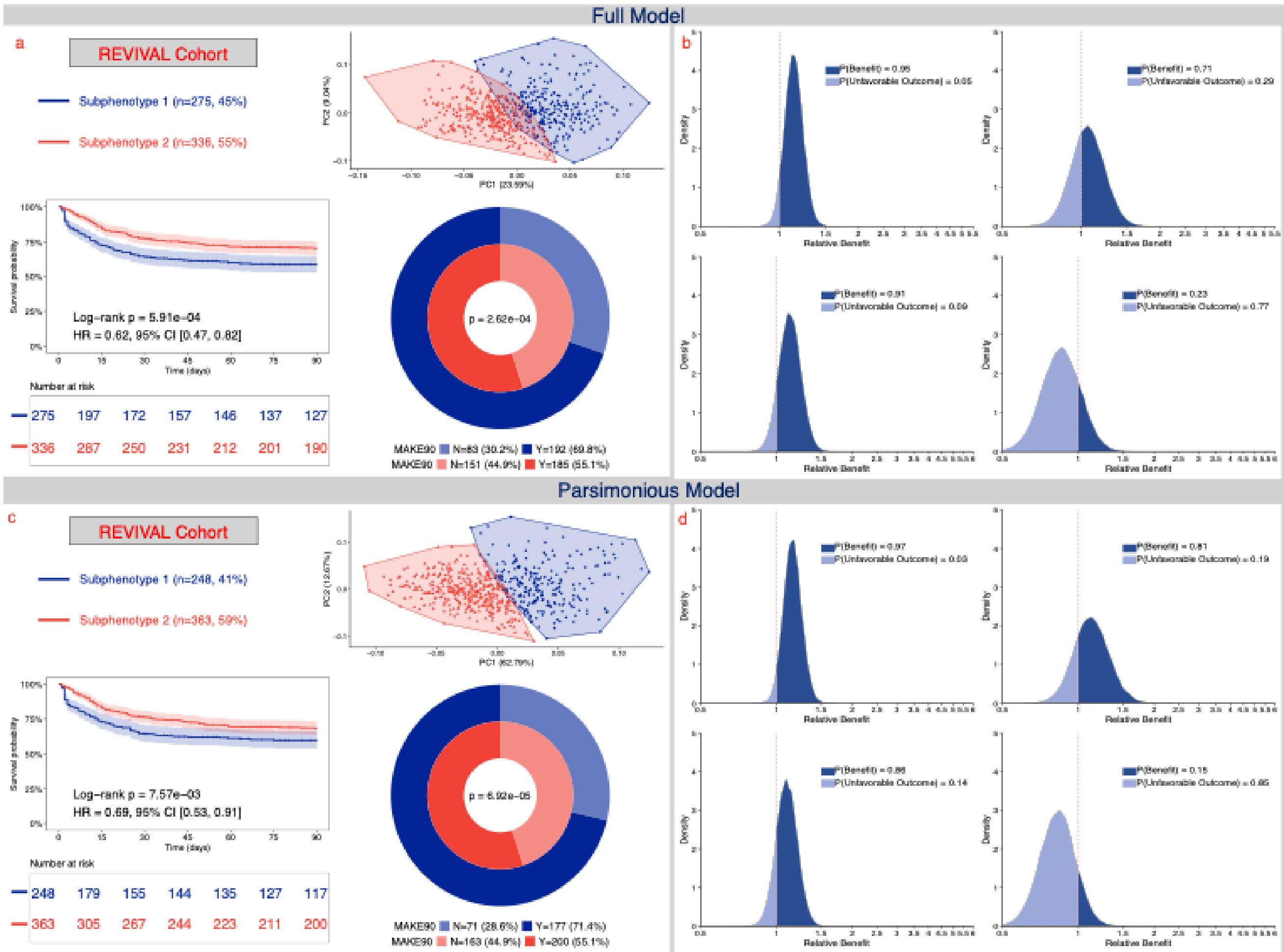

Panel a shows the full biomarker model results in the REVIVAL cohort (Subphenotype 1: n=275, 45%; Subphenotype 2: n=336, 55%). The Kaplan-Meier curve shows 90-day survival probability by subphenotype; log-rank test was two-sided ($p=5.91\times10^{-04}$). The hazard ratio was estimated using Cox proportional hazards regression (HR=0.62, 95% CI [0.47, 0.82]). Shaded bands represent 95% confidence intervals. The principal component analysis (PCA) plot shows proteomic separation between subphenotypes. The donut chart shows the distribution of Major Adverse Kidney Events at 90 days (MAKE90) by subphenotype; chi-square test was two-sided ($p=2.62\times10^{-04}$), where Y = having MAKE90 event by day 90 and N = no MAKE90 event by day 90. Panel b shows posterior density distributions of the relative benefit of recAP versus placebo from non-informative Bayesian logistic regression for the full model. Top row shows Subphenotype 1 results for MAKE90 (left) and 90-day mortality (right). Bottom row shows Subphenotype 2 results for MAKE90 (left) and 90-day mortality (right). The vertical dashed red line indicates relative risk (RR)=1. Dark blue shading represents P(Benefit); light blue shading represents P(Unfavorable Outcome). Panels c and d show the same analyses using the parsimonious 7-protein model (Subphenotype 1: n=248, 41%; Subphenotype 2: n=363, 59%; log-rank $p=7.57\times10^{-3}$; HR=0.69, 95% CI [0.53, 0.91]; donut chart $p=6.92\times10^{-05}$). Non-informative Bayesian logistic regression was used; no frequentist hypothesis testing was shown in this subfigure; results are presented as posterior probabilities only.

Supplementary Figure 3. Correlation of shared top 10% clustering driving proteins across cohorts.

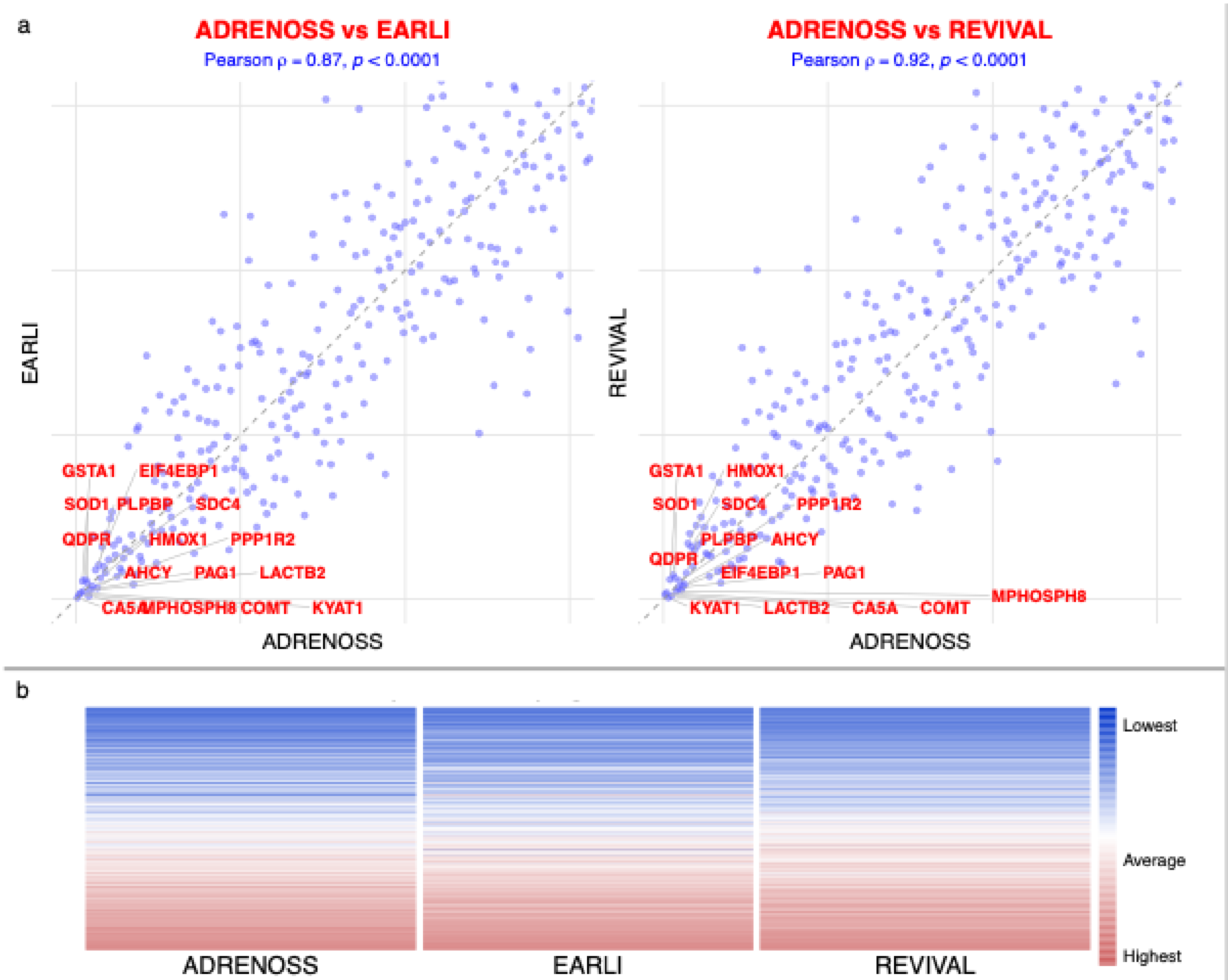

Panel a shows pairwise scatter plots of protein importance ranks between ADRENOSS and EARLI (left) and between ADRENOSS and REVIVAL (right), based on n = 359 proteins. Each point represents one protein; proteins labeled in red are shared in the top 10% of importance ranks across cohorts. Pearson correlation tests were two-sided with the null hypothesis that the true correlation is zero: ADRENOSS vs EARLI $r(357) = 0.87$ (95% CI [0.84, 0.89], df = 357); ADRENOSS vs REVIVAL $r(357) = 0.92$ (95% CI [0.90, 0.94], df = 357). No adjustments were made for multiple comparisons. Panel b shows a heatmap of importance ranks for n = 359 proteins across ADRENOSS, EARLI, and REVIVAL, where rows represent proteins and columns represent cohorts. Color scale ranges from lowest (dark blue) to highest (dark red) importance rank.

Supplementary Figure 4. Gene Ontology (GO) Biological Process enrichment of proteins upregulated in Subphenotype 2 across three cohorts: ADRENOSS, EARLI, REVIVAL.

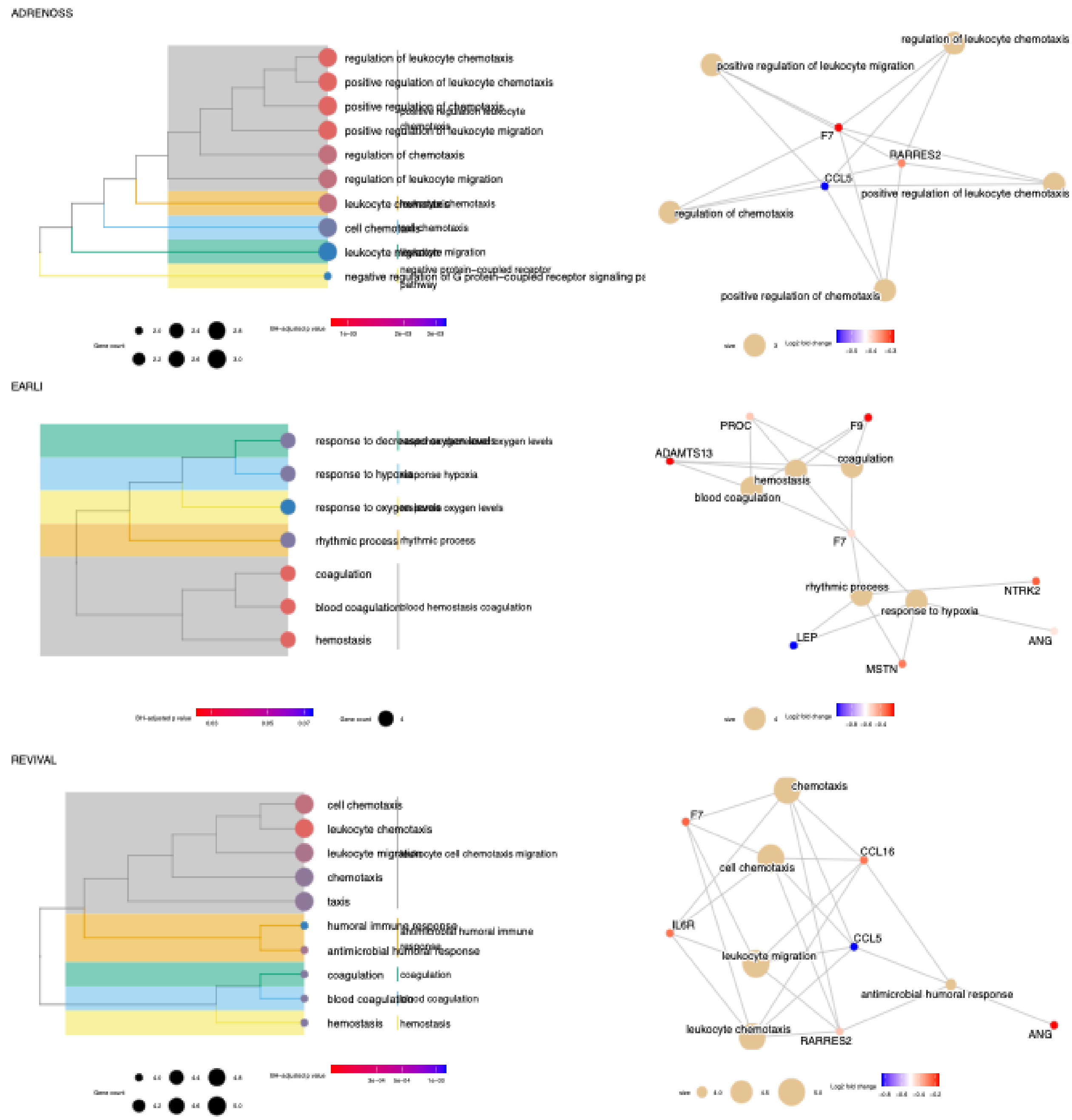

Subphenotype 2 (lower-risk subphenotype) in each cohort (ADRENOSS n = 7 proteins, EARLI n = 19 proteins, REVIVAL n = 13 proteins). In the treeplot, each node represents an enriched GO term, with node color indicating the Benjamini-Hochberg adjusted p-value (red = most significant, blue = least significant) and node size indicating the number of genes associated with the term. Related terms are grouped into clusters denoted by colored background bands. In the network plot, large beige nodes represent enriched GO terms and small colored nodes represent individual genes. Gene node color indicates Log2 fold change (Subphenotype 1 minus Subphenotype 2), where blue

indicates higher expression in Subphenotype 2. Node size reflects the number of genes per pathway. Edges connect genes to their associated pathways. The top 10 non-redundant GO terms are shown in the treeplot and the top 5 in the network plot, selected from a total of 27, 35, and 38 non-redundant GO terms in ADRENOSS, EARLI, and REVIVAL respectively. GO enrichment was performed using over-representation analysis with Benjamini-Hochberg correction; terms with FDR-adjusted p-value < 0.05 were considered significant. Over-representation analysis was one-sided, testing for enrichment only, with no additional adjustments beyond Benjamini-Hochberg correction applied within the enrichment analysis. Exact adjusted p-values for all enriched GO terms are provided in the Source Data file.

Supplementary Figure 5. Parsimonious model pipeline.

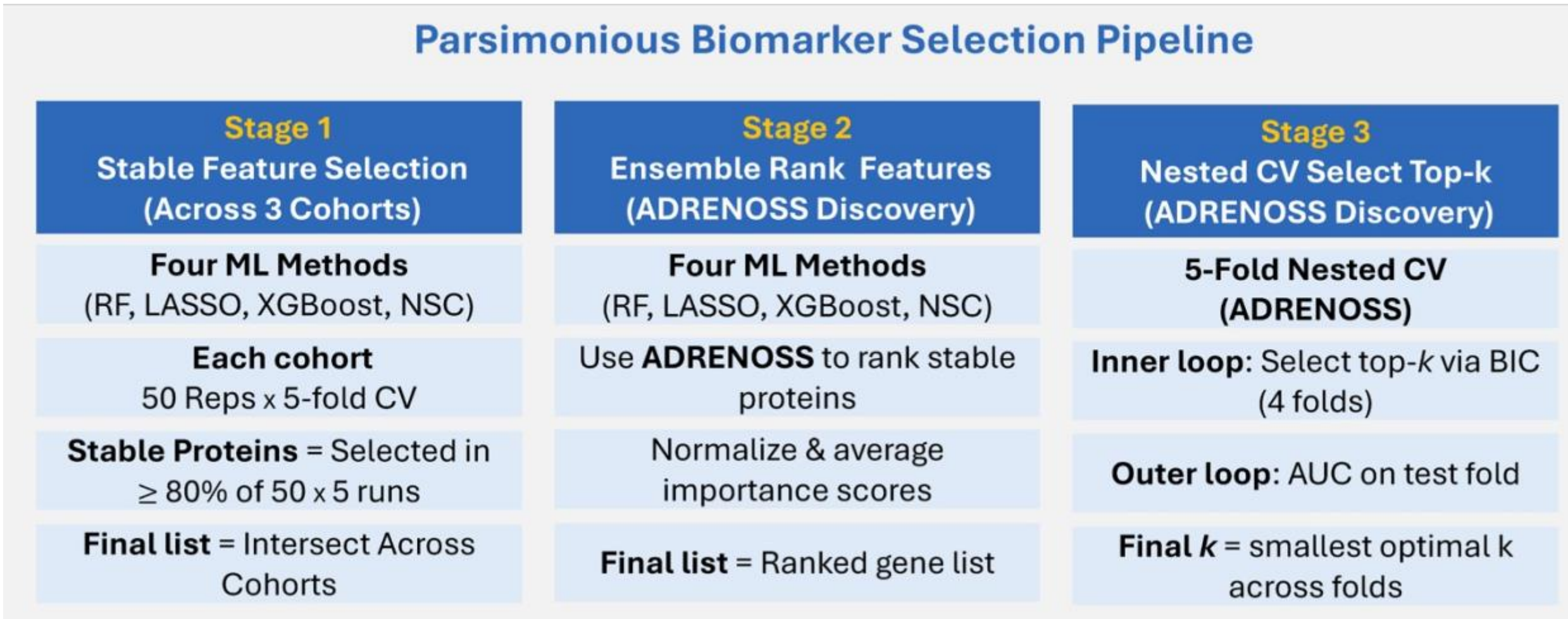

The three-stage parsimonious biomarker selection pipeline used to identify a reduced set of proteins for subphenotype across cohorts. Stage 1: Stable Feature Selection (across 3 cohorts): Four machine learning (ML) methods were applied: Random Forest (RF), Least Absolute Shrinkage and Selection Operator (LASSO), XGBoost, and Neural Sparse Coding (NSC). For each cohort, 50 repetitions of 5-fold cross-validation were performed. Proteins selected in ≥ 80% of 50×5 runs were considered stable. The final list was derived by taking the intersection of stable proteins across all three cohorts. Stage 2: Ensemble Rank Features (ADRENOSS Discovery): The same four ML methods were applied to the ADRENOSS discovery cohort to rank the stable proteins identified in Stage 1. Importance scores from each ML method were normalized and averaged to produce a final ranked protein list. Stage 3: Nested Cross-Validation Select Top-k (ADRENOSS Discovery): 5-fold nested cross-validation was used to select the optimal number of top-k proteins. In the inner loop (training folds), models were fit over a grid of k values, and the smallest Bayesian Information Criterion (BIC) was used to choose k. In the outer loop (held-out test fold), the chosen k was evaluated by area under the receiver operating characteristic curve (AUC). The final k was selected as the smallest k among the 5-fold-specific optimal values, resulting in the 7-protein parsimonious model.

Supplementary Figure 6. Comparison of upregulated (a) and downregulated (b) proteins between the full and parsimonious models in ADRENOSS, EARLI, and REVIVAL.

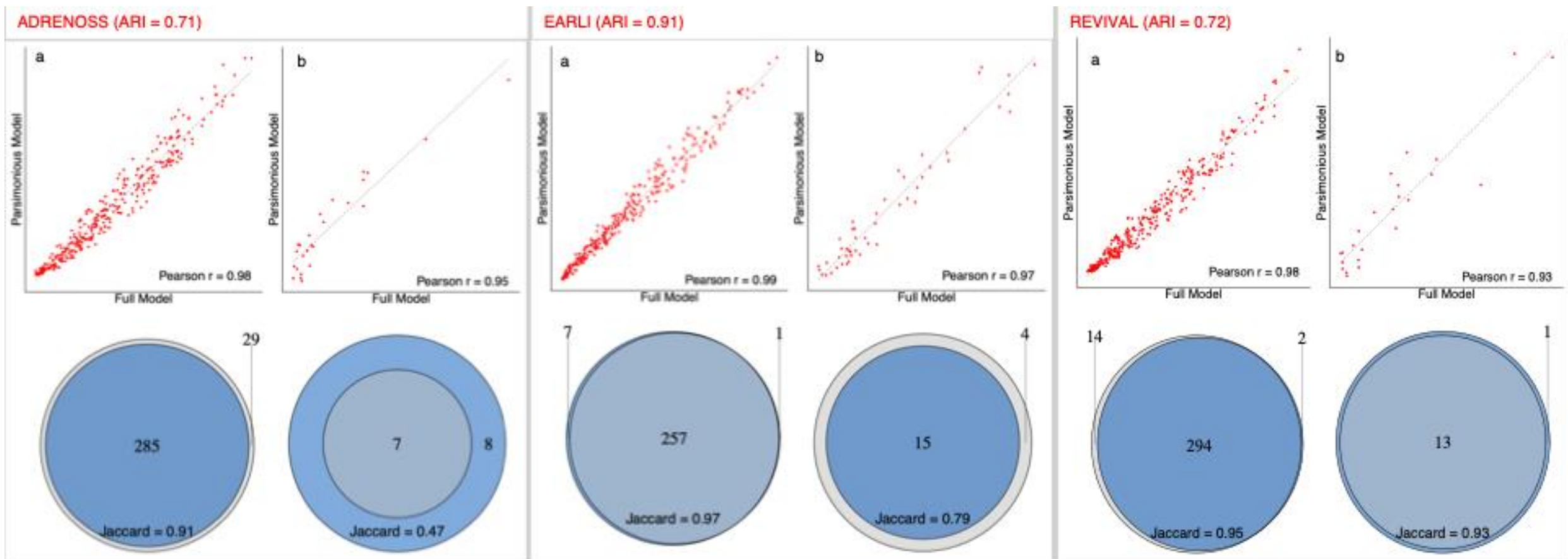

For each cohort, the Adjusted Rand Index (ARI) shown in the panel title quantifies the overall agreement in subphenotype assignments between the full and parsimonious models. Scatter plots show the concordance of protein statistical significance between the full biomarker model (x-axis) and the parsimonious 7-protein model (y-axis) for all proteins consistently upregulated in both models regardless of significance threshold (a; ADRENOSS n = 324, EARLI n = 308, REVIVAL n = 320) and consistently downregulated in both models (b; ADRENOSS n = 25, EARLI n = 49, REVIVAL n = 24). Each point represents one protein. The dashed line indicates perfect concordance. Pearson correlation tests were two-sided with no adjustments for multiple comparisons. Upregulated proteins: ADRENOSS $r(322) = 0.98$, $p = 1.46\times10^{-216}$, 95% CI [0.97, 0.98]; EARLI $r(306) = 0.99$, $p = 2.00\times10^{-248}$, 95% CI [0.98, 0.99]; REVIVAL $r(318) = 0.98$, $p = 5.72\times10^{-234}$, 95% CI [0.98, 0.99]. Downregulated proteins: ADRENOSS $r(23) = 0.95$, $p = 7.88\times10^{-13}$, 95% CI [0.88, 0.98]; EARLI $r(47) = 0.97$, $p = 1.28\times10^{-30}$, 95% CI [0.95, 0.98]; REVIVAL $r(22) = 0.93$, $p = 6.23\times10^{-11}$, 95% CI [0.84, 0.97]. Venn diagrams show the overlap of only significant proteins (adjusted p-value < 0.05) between the full model (gray) and parsimonious model (blue) for upregulated proteins (full model: ADRENOSS n = 314, EARLI n = 258, REVIVAL n = 308; parsimonious model: ADRENOSS n = 285, EARLI n = 264, REVIVAL n = 296) and downregulated proteins (full model: ADRENOSS n = 7, EARLI n = 19, REVIVAL n = 13; parsimonious model: ADRENOSS n = 15, EARLI n = 15, REVIVAL n = 14). Jaccard index measures the similarity between the two protein sets, calculated as the size of the intersection divided by the size of the union.

Supplementary Figure 7. Bayesian A/B test results for treatment effects on MAKE90 and DEAD90 in the REVIVAL cohort.

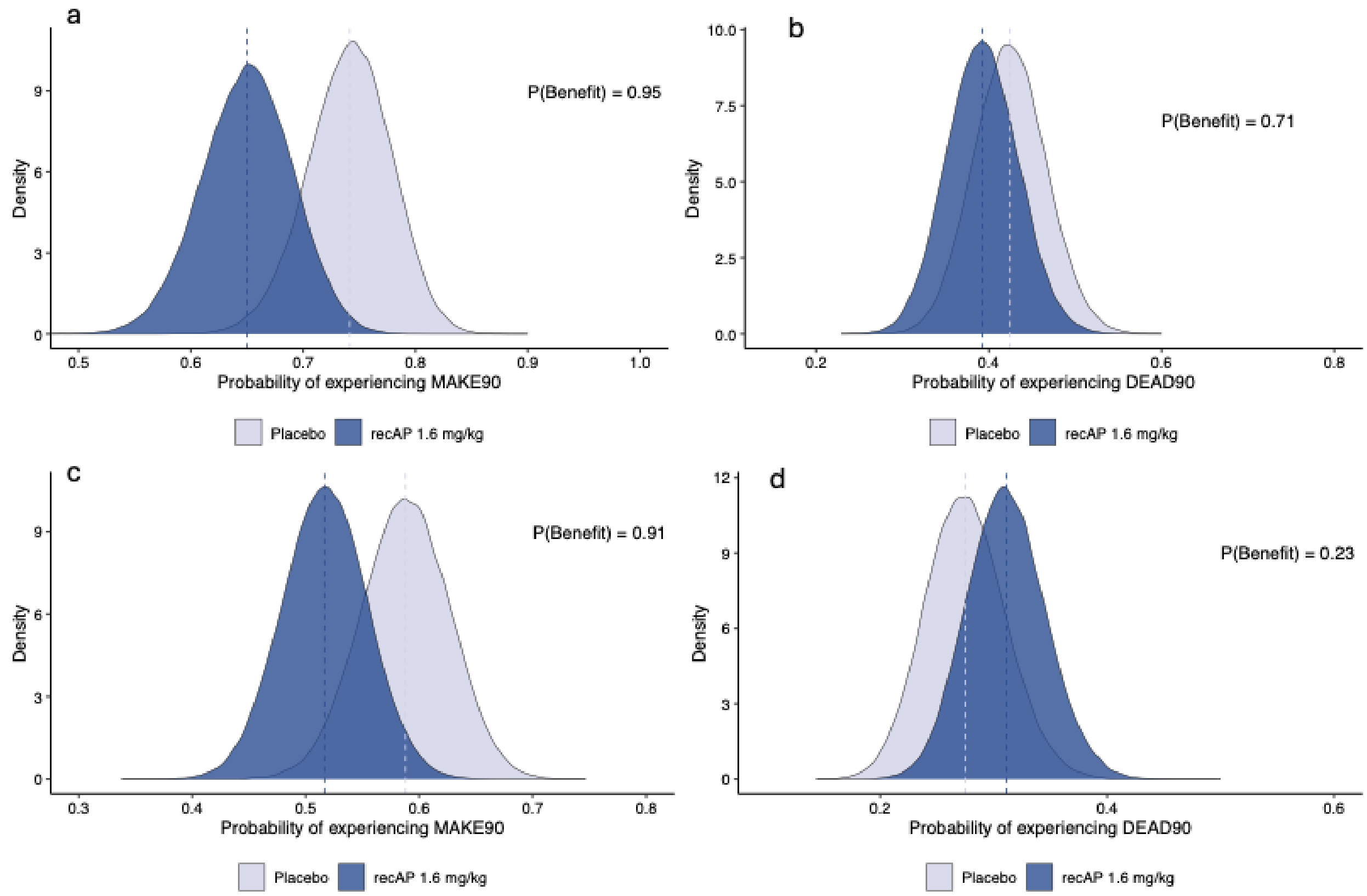

Posterior probability distributions of experiencing MAKE90 and DEAD90 for recombinant alkaline phosphatase (recAP, 1.6 mg/kg) versus placebo in the REVIVAL cohort, stratified by subphenotype. Panels a and b show results for Subphenotype 1 (placebo n=137, recAP n=138) for MAKE90 and DEAD90 respectively. Panels c and d show results for Subphenotype 2 (placebo n=158, recAP n=178) for MAKE90 and DEAD90 respectively. Analyses used a Bayesian A/B test with Bernoulli likelihood and non-informative Beta(1,1) priors. P(Benefit) represents the posterior probability that recAP reduces the risk of the outcome compared to placebo, defined as P(probability of outcome in recAP group < probability of outcome in placebo group). Vertical dashed lines indicate posterior mean probability for each group. Dark blue shading represents the recAP 1.6 mg/kg group; light blue shading represents the placebo group. Non-informative Bayesian logistic regression was used, and results are presented as posterior probabilities only.

Supplementary Figure 8. Bayesian A/B test results for treatment effects within Subphenotypes in the REVIVAL parsimonious model.

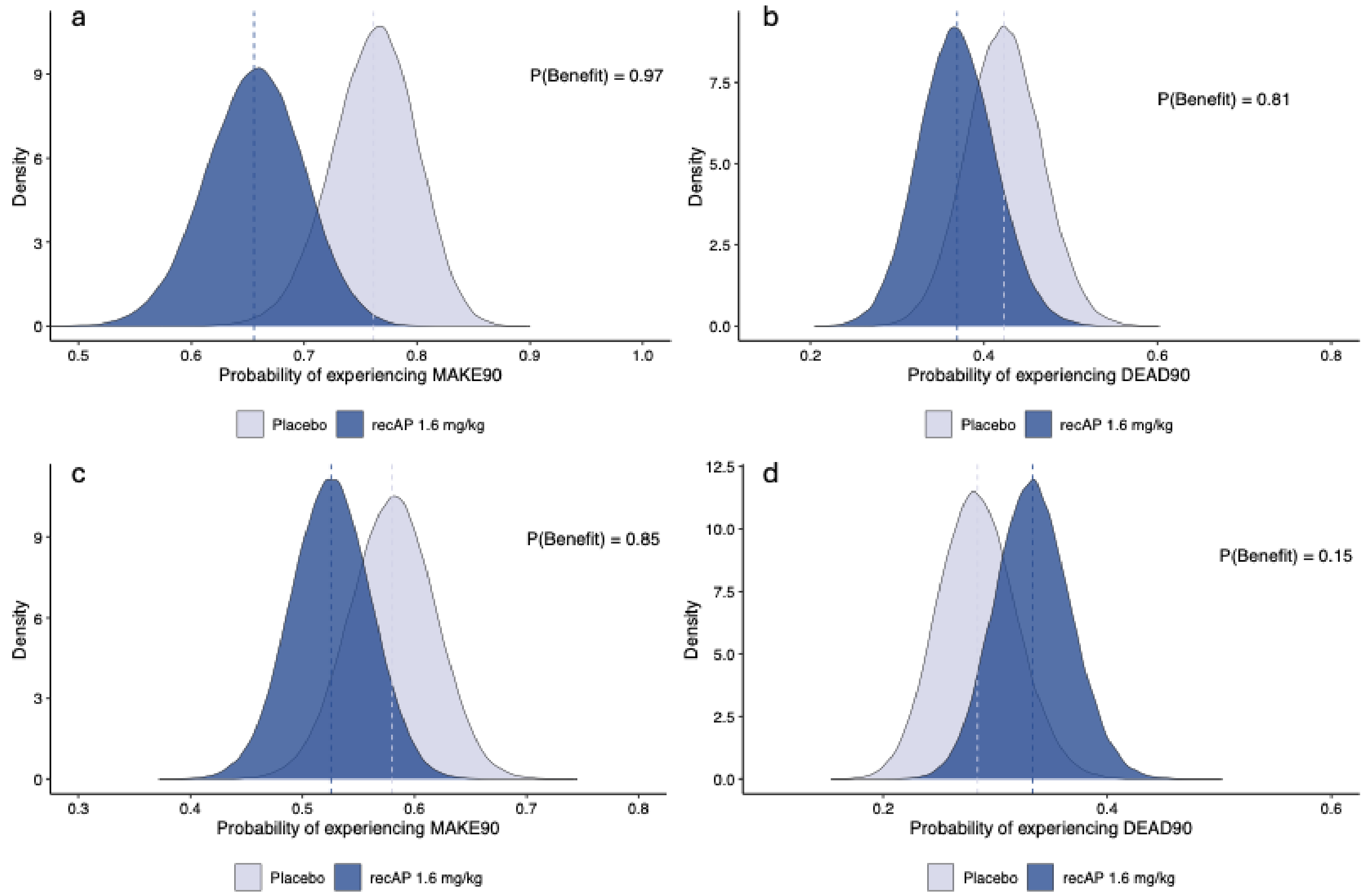

Posterior probability distributions of experiencing MAKE90 and DEAD90 for recombinant alkaline phosphatase (recAP, 1.6 mg/kg) versus placebo in the REVIVAL cohort, stratified by subphenotype using the parsimonious 7-protein model. Panels a and b show results for Subphenotype 1 (placebo n=128, recAP n=120) for MAKE90 and DEAD90 respectively. Panels c and d show results for Subphenotype 2 (placebo n=167, recAP n=196) for MAKE90 and DEAD90 respectively. Analyses used a Bayesian A/B test with Bernoulli likelihood and non-informative Beta(1,1) priors. P(Benefit) represents the posterior probability that recAP reduces the risk of the outcome compared to placebo, defined as P(probability of outcome in recAP group < probability of outcome in placebo group). Vertical dashed lines indicate posterior mean probability for each group. Dark blue shading represents the recAP 1.6 mg/kg group; light blue shading represents the placebo group. Non-informative Bayesian logistic regression was used, and results are presented as posterior probabilities only.

Supplementary Figure 9. Cell-of-origin of the plasma proteins.

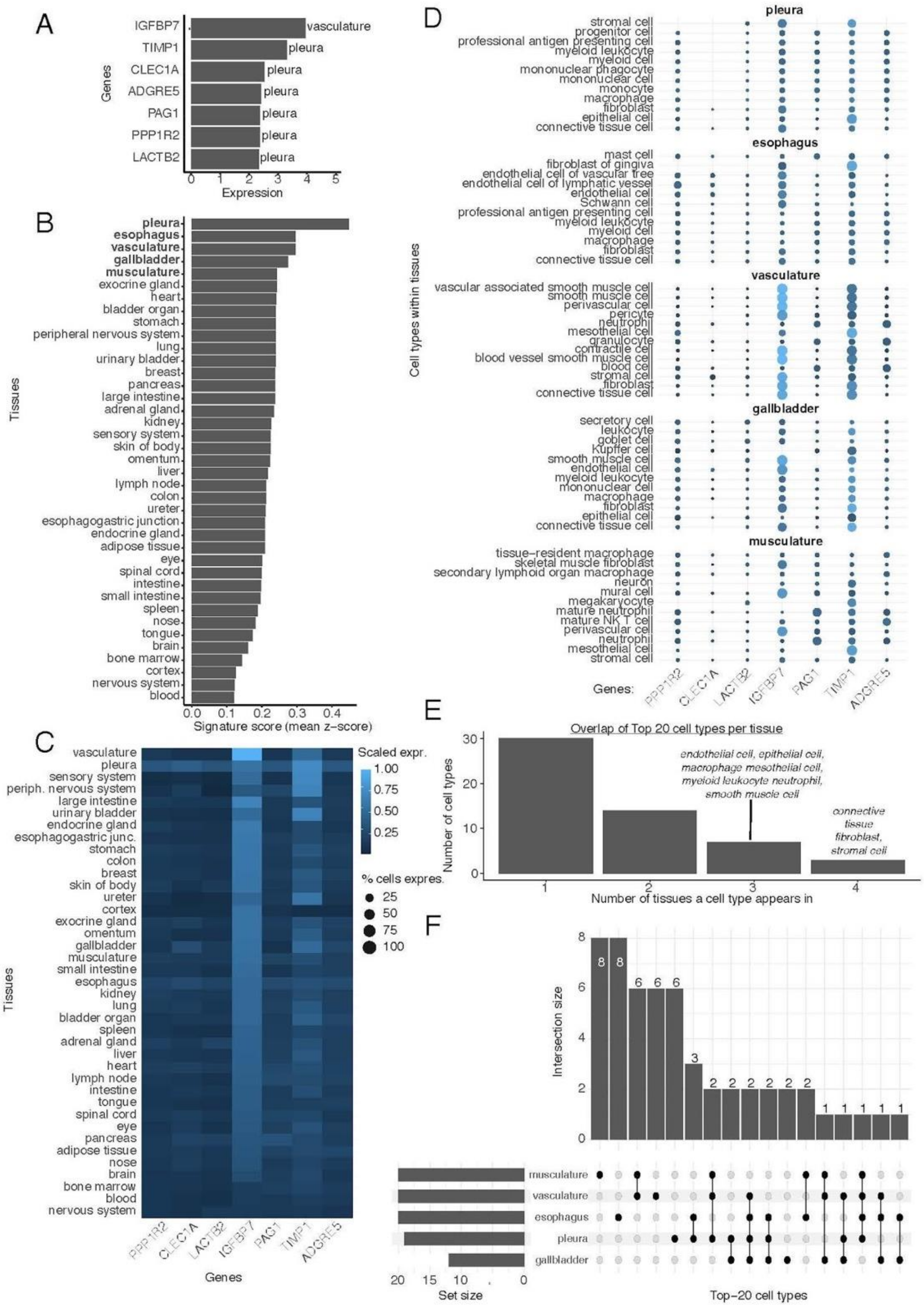

Supplementary Figure 10. Validation of ADRENOSS - classification model pipeline.

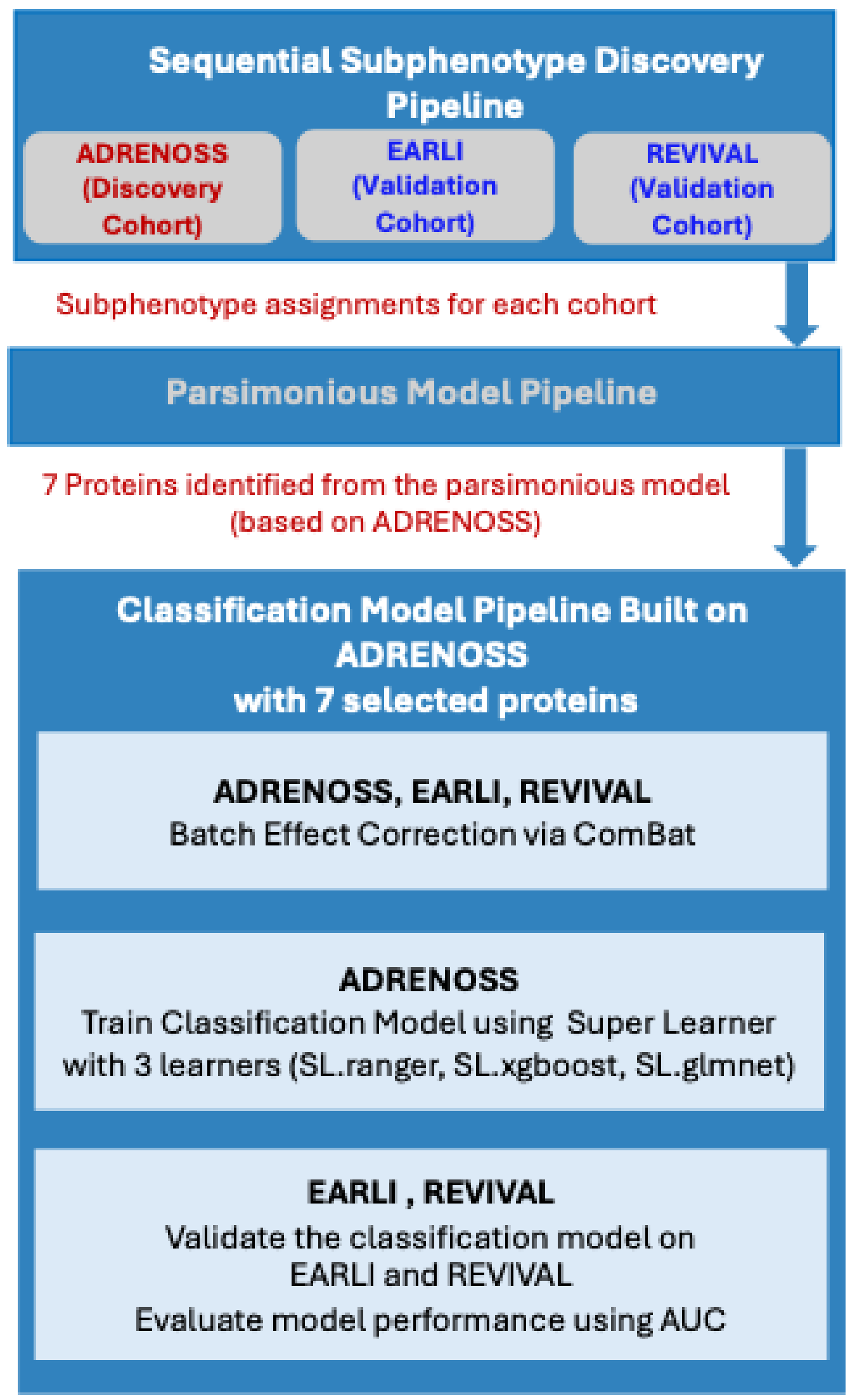

The sequential pipeline used to derive and validate subphenotype assignments across three cohorts and to build a parsimonious classification model. After the Sequential Subphenotype Discovery Pipeline generating the subphenotype assignments for each cohort - ADRENOSS, EARLI, REVIVAL- the Parsimonious Model Pipeline (Supplementary Figure 1) was applied to identify 7 proteins from the ADRENOSS discovery cohort. Then, a Classification Model Pipeline was built using these 7 selected proteins in three steps: (1) batch effect correction was performed across ADRENOSS, EARLI, and REVIVAL using ComBat; (2) a Super Learner classification model was trained on ADRENOSS using three base learners: SL.ranger, SL.xgboost, and SL.glmnet; (3) the trained model was validated on EARLI and REVIVAL and model performance was evaluated using the area under the receiver operating characteristic curve (AUC).

Supplementary Tables

Supplementary Table 1. Tables of shared top 10% proteins from the cluster driving partition.

		Variable Importance Ranking		
**Gene Name**	**Protein Name**	**ADRENOSS**	**EARLI**	**REVIVAL**
CA5A	Carbonic anhydrase 5A, mitochondrial	1	1	3
COMT	Catechol O-methyltransferase	2	3	4
KYAT1	Kynurenine--oxoglutarate transaminase 1	3	4	1
GSTA1	Glutathione S-transferase A1	4	11	12
LACTB2	Endoribonuclease LACTB2	5	5	2
QDPR	Dihydropteridine reductase	6	12	15
SOD1	Superoxide dismutase [Cu-Zn]	7	10	13
MPHOSPH8	M-phase phosphoprotein 8	8	2	5
PAG1	Phosphoprotein associated with glycosphingolipid-enriched microdomains 1	9	6	7
PPP1R2	Protein phosphatase inhibitor 2	10	8	11
EIF4EBP1	Eukaryotic translation initiation factor 4E-binding protein 1	12	18	8
AHCY	Adenosylhomocysteinase	13	7	9
PLPBP	Pyridoxal phosphate homeostasis protein	14	21	16
HMOX1	Heme oxygenase 1	15	13	34
SDC4	Syndecan-4	17	20	32
TYMP	Thymidine phosphorylase	18	16	20
GLRX	Glutaredoxin-1	24	27	35
CLEC5A	C-type lectin domain family 5 member A	26	36	28
CES1	Liver carboxylesterase 1	27	19	27
FGFR1OP	FGFR1 oncogene partner	28	17	14
FBP1	Fructose-1,6-bisphosphatase 1	29	25	26
DIABLO	Diablo homolog, mitochondrial	33	29	17
RCOR1	REST corepressor 1	35	9	10
ZBTB17	Zinc finger and BTB domain-containing protein 17	36	15	18

Supplementary Table 2. Cross-Cohort Shared Top 10% Upregulated Proteins in Subphenotype 1

UniProt	Protein Name	Log Fold Change		
		ADRENOSS	EARLI	REVIVAL
O94903	Pyridoxal phosphate homeostasis protein	1.83	2.05	1.89
P00441	Superoxide dismutase [Cu-Zn]	1.82	1.93	1.71
P08263	Glutathione S-transferase A1	2.94	3.24	2.94
P09417	Dihydropteridine reductase	1.99	2.09	1.89
P09467	Fructose-1,6-bisphosphatase 1	2.14	2.76	2.68
P09601	Heme oxygenase 1	1.79	2.17	1.68
P10145	Interleukin-8	2.22	3.11	2.20
P17516	Aldo-keto reductase family 1 member C4	1.54	1.78	2.36
P19971	Thymidine phosphorylase	1.55	1.95	1.77
P20711	Aromatic-L-amino-acid decarboxylase	1.97	1.96	2.02
P21549	Serine--pyruvate aminotransferase	1.81	1.95	1.79
P21964	Catechol O-methyltransferase	2.32	2.42	2.19
P23141	Liver carboxylesterase 1	1.96	2.68	2.18
P23526	Adenosylhomocysteinase	2.11	2.40	2.41
P31431	Syndecan-4	1.73	2.04	1.61
P35218	Carbonic anhydrase 5A, mitochondrial	3.77	3.92	3.45
P41236	Protein phosphatase inhibitor 2	1.59	1.83	1.73
Q13541	Eukaryotic translation initiation factor 4E-binding protein 1	1.87	2.17	2.03
Q16773	Kynurenine--oxoglutarate transaminase 1	2.55	2.74	2.61
Q53H82	Endoribonuclease LACTB2	2.14	2.27	2.23
Q99549	M-phase phosphoprotein 8	2.07	2.39	2.14
Q9NR28	Diablo homolog, mitochondrial	1.84	1.84	2.37
Q9NWQ8	Phosphoprotein associated with glycosphingolipid-enriched microdomains 1	1.73	2.00	1.80
Q9UKL0	REST corepressor 1	1.55	2.17	2.14

Supplementary Table 3. Detailed MAKE90 components across three cohorts:

	ADRENOSS					EARLI					REVIVAL				
	All N = 319[1]	Sub. 1 N = 132[1]	Sub. 2 N = 187[1]	P-value[2]	Q-value[3]	All N = 169[1]	Sub. 1 N = 59[1]	Sub. 2 N = 110[1]	P-value[2]	Q-value[3]	All N = 611[1]	Sub. 1 N = 275[1]	Sub. 2 N = 336[1]	P-value[2]	Q-value[3]
**MAKE90**	209 (68%)	110 (85%)	99 (56%)	<0.001	<0.001	92 (54%)	47 (80%)	45 (41%)	<0.001	<0.001	377 (62%)	192 (70%)	185 (55%)	<0.001	0.001
**Death within 90 Days**	122 (38%)	69 (52%)	53 (28%)	<0.001	<0.001	81 (48%)	44 (75%)	37 (34%)	<0.001	<0.001	210 (34%)	112 (41%)	98 (29%)	0.004	0.011
**RRT within 90 days**	72 (23%)	48 (36%)	24 (13%)	0.002	0.002	36 (21%)	26 (44%)	10 (9%)	<0.001	<0.001	201 (33%)	129 (47%)	72 (21%)	<0.001	<0.001
**≥25% decline in eGFR from baseline**	142 (51%)	78 (67%)	64 (39%)	<0.001	<0.001	35 (21%)	15 (25%)	20 (18%)	0.4	0.4	45 (7%)	15 (5%)	30 (9%)	0.14	0.3

[1]n (%); Mean (SD)
[2]Welch two-sample t-test (two-sided) for continuous variables; Pearson's chi-squared test or Fisher's exact test (two-sided) for categorical variables (Fisher's exact test used when expected cell count < 5)
[3]Holm correction for multiple testing
Sub. = subphenotype

Supplementary Table 4. List of the 7 proteins used in the parsimonious model.

Protein	Gene Symbol	Function / Expression	Prior evidence in AKI & Sepsis
**Protein phosphatase inhibitor 2**	PPP1R2	Regulates PP1 activity; widely expressed in muscle and nervous tissue	Not documented
**C-type lectin domain family 1 member A**	CLEC1A	Immune recognition receptor; expressed on dendritic cells and endothelial cells	Not documented
**Endoribonuclease LACTB2**	LACTB2	Mitochondrial RNA processing; moderate expression across tissues	Not documented
**Insulin-like growth factor-binding protein 7 (IGFBP7)**	IGFBP7	Binds IGFs, modulates their bioavailability; expressed in renal tubules, vasculature	**AKI biomarker** (part of [TIMP2] ×[IGFBP7]); prognostic in sepsis-associated AKI
**Phosphoprotein associated with glycosphingolipid-enriched microdomains 1 (PAG1)**	PAG1	Scaffold protein in lipid rafts; regulates T-cell signaling; expressed in immune and epithelial tissues	Not documented
**Tissue inhibitor of metalloproteinases 1 (TIMP1)**	TIMP1	Inhibits MMPs, expressed ubiquitously including liver, bone marrow, fibroblasts, endothelial cells.	**AKI biomarker**: has been found elevated in AKI I human and animal models
**CD97 antigen (adhesion G protein-coupled receptor E5)**	CD97	Cell adhesion and migration; expressed on leukocytes and epithelia; low kidney expression	Not documented

Supplementary Table 5. Clinical Outcomes by Cluster Across Three Cohorts: Parsimonious + Clinical vs. Parsimonious Models

		Parsimonious + Clinical				Parsimonious Only			
**ADRENOSS**	**Outcomes**	**Sub. 1 N = 131[1]**	**Sub. 2 N=188[1]**	**P-value[2]**	**Q-value**	**Sub. 1 N=131[1]**	**Sub. 2 N=188[1]**	**P-value[2]**	**Q-value**
	Death within 28 days	56 (43%)	38 (20%)	<0.001	<0.001	59 (45%)	35 (19%)	<0.001	<0.001
	Death within 90days	67 (51%)	55 (29%)	<0.001	<0.001	69 (53%)	53 (28%)	<0.001	<0.001
	MAKE90	109 (84%)	100 (57%)	<0.001	<0.001	108 (84%)	101 (57%)	<0.001	<0.001
	ICU length of stay	5 [3-11]	6 [4-11]	0.4	0.4	6 [3-13]	6 [3-10]	0.6	0.6
	28-day ICU-free days	0 [0-22]	20 [0-24]	<0.001	<0.001	0 [0-21]	21 [5-24]	<0.001	<0.001
	90-day ICU-free days	0 [0-83]	81 [0-86]	<0.001	<0.001	0 [0-82]	82 [0-86]	<0.001	<0.001
**EARLI**	**Outcomes**	**Sub. 1 N = 67[1]**	**Sub. 2 N=102[1]**	**P-value[2]**	**Q-value**	**Sub. 1 N=59[1]**	**Sub. 2 N=110[1]**	**P-value[2]**	**Q-value**
	Death within 28 days	42 (63%)	32 (31%)	<0.001	<0.001	38 (64%)	36 (33%)	<0.001	<0.001
	Death within 90days	46 (69%)	35 (34%)	<0.001	<0.001	42 (71%)	39 (35%)	<0.001	<0.001
	MAKE90	51 (76%)	41 (40%)	<0.001	<0.001	45 (76%)	47 (43%)	<0.001	<0.001
	ICU length of stay	6 [4-11]	5 [3-7]	0.11	0.11	7 [4-11]	5 [3-8]	0.2	0.2
	28-day ICU-free days	0 [0-14]	21 [0-24]	<0.001	<0.001	0 [0-13]	20 [0-24]	<0.001	<0.001
	90-day ICU-free days	0 [0-71]	82 [0-86]	<0.001	<0.001	0 [0-70]	80 [0-86]	<0.001	<0.001
**REVIVAL**	**Outcomes**	**Sub. 1 N = 276[1]**	**Sub. 2 N=335[1]**	**P-value[2]**	**Q-value**	**Sub. 1 N=248[1]**	**Sub.2 N=363[1]**	**P-value[2]**	**Q-value**
	Death within 28 days	98 (36%)	74 (22%)	<0.001	0.001	87 (35%)	85 (23%)	0.002	0.007
	Death within 90days	114 (48%)	96 (33%)	0.001	0.003	98 (46%)	112 (36%)	0.032	0.065
	MAKE90	126 (53%)	109 (40%)	0.003	0.003	107 (51%)	128 (42%)	0.063	0.065
	28-day ICU-free days	0 [0-18]	18 [0-23]	<0.001	<0.001	0 [0-18]	17 [0-23]	<0.001	<0.001
	90-day ICU-free days	30 [0-77]	76 [0-85]	<0.001	<0.001	38 [0-77]	74 [0-84]	<0.001	<0.001

[1]n (%); Mean (SD); Median [Q1-Q2]
[2]Welch two-sample t-test (two-sided) for continuous variables; Pearson's chi-squared test or Fisher's exact test (two-sided) for categorical variables (Fisher's exact test used when expected cell count < 5)
[3]Holm correction for multiple testing
Sub. = Subphenotype

Supplementary Table 6. Detailed treatment effect by cluster of recombinant alkaline phosphatase (recap, ilofotase alfa) in the REVIVAL trial.

	Subphenotype 1				Subphenotype 2			
	Overall[1] N = 275	Placebo[1] N = 137	recAP 1.6 mg/kg [1] N = 138	P-value[2]	Overall[1] N = 336	Placebo[1] N = 158	recAP 1.6 mg/kg[1] N = 178	P-value[2]
**28-Day Mortality**	97 (35%)	51 (37%)	46 (33%)	0.5	75 (22%)	31 (20%)	44 (25%)	0.3
**90-Day Mortality**	112 (41%)	58 (42%)	54 (39%)	0.6	98 (29%)	43 (27%)	55 (31%)	0.5
**180-Day Mortality**	119 (43%)	61 (45%)	58 (42%)	0.8	104 (31%)	44 (28%)	60 (34%)	0.2
**28-day ICU-free days**	8.1 (9.5)	8.2 (9.5)	8.0 (9.5)	0.9	13.6 (10.6)	13.1 (10.9)	14.1 (10.3)	0.8
**MAKE90**	192 (70%)	102 (74%)	90 (65%)	0.1	185 (45%)	93 (59%)	92 (52%)	0.2

[1]*n (%); Mean (SD)*

[2]*Welch two-sample t-test (two-sided) for continuous variables; Pearson's chi-squared test or Fisher's exact test (two-sided) for categorical variables (Fisher's exact test used when expected cell count < 5)*

Supplementary Table 7. Bayesian Logistic Regression for HTE with interaction terms in REVIVAL

Outcome	Interaction OR (Subphenotye 1 vs 2; recap 1.6mg/kg vs Placebo) Median (90% CI)	P (OR < 0.95)	P (OR > 1.05)
**Full Model**			
MAKE90A	0.86 (0.48–1.51)	0.62	0.28
DEAD90	0.73 (0.41–1.29)	0.78	0.15
**Parsimonious Model**			
MAKE90A	0.74 (0.41–1.31)	0.76	0.16
DEAD90	0.62 (0.35–1.10)	0.89	0.07

Outcomes are adverse when =1;
OR < 1 indicates lower odds of adverse outcome in Subphenotype 1 (greater benefit)
P(OR<0.95) = Posterior probabilities that the treatment OR in subphenotype 1 is at least 5% lower than then treatment OR in subphenotype 2.

P(OR>1.05) = Posterior probabilities that the treatment OR in subphenotype 1 is at least 5% higher than then treatment OR in subphenotype 2.

Supplementary Table 8. Outcome associations by subphenotype in ADRENOSS patients after inclusion of healthy control samples in the clustering framework (n=319 patients; n=108 healthy controls).

	**Parsimonious 7-Biomarker Model**				**Full 361-Biomarker Model**			
	**Subphenotype 1**	**Subphenotype 2**	**P-value**[2]	**Q-value**[3]	**Subphenotype 1**	**Subphenotype 2**	**P-value**[2]	**Q-value**[3]
	N = 134[1]	N = 185[1]			N = 199[1]	N = 120[1]		
**28-Day Mortality**	59 (44%)	35 (19%)	<0.001	<0.001	76 (38%)	18 (15%)	<0.001	<0.001
**90-Day Mortality**	69 (51%)	53 (29%)	<0.001	<0.001	94 (47%)	28 (23%)	<0.001	<0.001
**MAKE within 90 Days**	109 (83%)	100 (57%)	<0.001	<0.001	159 (81%)	50 (45%)	<0.001	<0.001

[1]*n (%); Mean (SD)*

[2]*Welch two-sample t-test (two-sided) for continuous variables; Pearson's chi-squared test or Fisher's exact test (two-sided) for categorical variables (Fisher's exact test used when expected cell count < 5)*

[3]*Holm correction for multiple testing*

Outcome analysis restricted to ADRENOSS patients (n=319). Healthy control samples (n=108) were included in the clustering framework to assess biological plausibility but do not have clinical outcome data and are therefore excluded from this table.

Supplement Results: Sensitivity and Ancillary Analyses

Evaluation Using Healthy Control Samples

Classification model: Using the seven-biomarker parsimonious model, 105 (97.2%) healthy samples were assigned to the lower-risk subphenotype, with three classified as higher-risk. Using the full biomarker model, 104 (96.3%) were assigned to the lower-risk subphenotype and four to the higher-risk subphenotype.

Unsupervised clustering: Under the full biomarker model applied to the combined ADRENOSS and healthy samples (n = 427), all healthy samples were assigned to the lower-risk subphenotype, with no healthy samples in the higher-risk group. Under the seven-biomarker parsimonious model, 106 (98.2%) healthy samples were clustered with the lower-risk subphenotype and two with the higher-risk group.

Inclusion of healthy samples resulted in limited reassignment among ADRENOSS patients, and associations with primary outcomes were consistent with the primary analysis (Supplementary Table 8). Together, both approaches confirm that healthy individuals align with the lower-risk subphenotype.

Early (Within 48-Hour) Sepsis-Associated Acute Kidney Injury (SAKI)

In both ADRENOSS and EARLI, a two-subphenotype structure was again identified with the lowest PAC when clustering was restricted to patients with AKI within 48 hours. Biomarker ranking derived from the 48-hour AKI analysis was consistent with those from the 7-day AKI analysis: ADRENOSS Spearman rho (df = 357) = 0.83, $p = 4.49\times10^{-93}$, 95% CI [0.80, 0.86]; EARLI Spearman rho (df = 357) = 0.93, $p = 1.03\times10^{-123}$, 95% CI [0.91, 0.94]. These findings suggest that subphenotype structure and biomarker importance were not substantially altered by inclusion of later-onset AKI cases.

Comparison with Angiopoietin/TNFR Subphenotypes

The AUC for predicting clustering assignments from Ang2/Ang1 and sTNFR1 was 0.669 for the full model clusters and 0.655 for the parsimonious clusters, indicating modest discriminatory ability. Agreement between clusters derived from Ang1, Ang2, and sTNFR1 and those derived from the parsimonious biomarker panel was low (Jaccard index = 0.32; adjusted Rand index = 0.15).

Incorporation of Clinical Variables

Agreement between biomarker-only and biomarker-plus-clinical-variable clustering assignments was high across cohorts (parsimonious panel: ADRENOSS 0.87, EARLI 0.88, REVIVAL 0.80; full panel: ADRENOSS 0.80, EARLI 0.80, REVIVAL 0.82), indicating that the original subphenotype structure was largely maintained. Predictive performance of clinical variables alone for biomarker-derived cluster membership was modest (parsimonious clusters: AUC 0.68, 0.78, and 0.64 for ADRENOSS, EARLI, and REVIVAL, respectively; full-model clusters: AUC 0.67, 0.79, and 0.65). Adding clinical variables to the parsimonious biomarker panel did not meaningfully

improve external prediction performance (EARLI AUC 0.995 vs. 0.995; REVIVAL AUC 0.984 vs. 0.984). Together, these analyses show that the identified subphenotypes are primarily driven by biomarker patterns rather than demographic or clinical characteristics.

Cluster Stability and Sample Size Considerations

In all three cohorts, the two-cluster solution yielded the lowest PAC, indicating the most stable partition under resampling. In the enlarged pooled dataset of 1,099 patients, the two-cluster solution again demonstrated the lowest PAC across all evaluated values of k. These findings suggest that the two-subphenotype structure is stable under resampling and is not materially altered by moderate increases in sample size within the range studied.

Frequentist Evaluation of Treatment Effects

In Cluster 1, the hazard ratio (HR) for recAP versus placebo was 0.87 (Wald $z = -0.721$, $df = 1$, $p = 0.471$, 95% CI [0.60, 1.26]). In Cluster 2, the HR was 1.15 (Wald $z = 0.705$, $df = 1$, $p = 0.481$, 95% CI [0.77, 1.72]). In a model including a treatment-by-subphenotype interaction term, the interaction HR was 1.33 (Wald $z = 1.029$, $df = 1$, $p = 0.304$, 95% CI [0.77, 2.29]), indicating no statistically significant evidence of differential treatment effect between subphenotypes.
