# Supplementary material for: Plasma proteomics defines two reproducible subphenotypes of sepsis-associated acute kidney injury with distinct outcomes
Source: Nat Commun. 2026 Aug 19;17:10575. doi: 10.1038/s41467-026-76889-3 (PMC13646287; doi:10.1038/s41467-026-76889-3)
Supplement: Supplementary file 2 [file 41467_2026_76889_MOESM2_ESM.pdf]

nature portfolio

Peer Review File

Plasma proteomics defines two reproducible subphenotypes of sepsis-associated acute kidney injury with distinct outcomes

Corresponding Author: Professor Matthieu LEGRAND

This file contains all reviewer reports in order by version, followed by all author rebuttals in order by version.

Version 0:

Reviewer comments:

Reviewer #1

(Remarks to the Author)
In the study by Legrand et al., the authors performed a plasma proteomics analysis on patients with sepsis-associated acute kidney injury (SA-AKI). They identified two major patient clusters that were consistently associated with sepsis-related mortality and survival across three independent clinical trials (ADRENOSS, n = 319; EARLI, n = 169; REVIVAL, n = 611). Subsequently, they developed a seven-protein predictive model to classify patients into these clusters. Notably, Bayesian modeling applied to the REVIVAL cohort suggested stronger discriminative performance among patients treated with ilofotase alfa. Overall, the findings are compelling and highlight potential biomarker targets for patient stratification and therapeutic response prediction in sepsis-associated kidney injury.

Major Comments
1. The original EARLI study reportedly included 818 samples, yet only 169 were analyzed in the present work. The criteria for this subset selection remain unclear—clarification is needed regarding why only approximately 20% of available samples were used.
2. Given that the VALID trial cohort might be available, does the proteomics clustering and seven-protein model be validated in the VALID cohort?
3. Question on cell-of-origin of the plasma protein. Beyond pathway enrichment results, it would be valuable to explore the cell of origin and tissue sources of the seven identified proteins. Identifying which organs predominantly secrete these proteins could enhance the biological interpretability of the clustering and its relation to organ-specific pathophysiology.
4. Question on data processing. The reviewer dataset appears to contain scaled or normalized values (including negative numbers). The manuscript should clarify the preprocessing steps applied to the proteomics data and discuss whether new patient samples can be normalized and integrated consistently into the existing model framework when predicting cluster membership.
5. Inclusion of healthy control. As part of model validation, it would be informative to assess the seven-protein model using normal plasma samples (if available). In principle, such samples should cluster with the lower-risk group, providing an important biological sanity check for the model.
6. Timing of plasma collection. Since plasma samples were collected at study enrollment in each trial, it is important to clarify whether the timing of sample collection was comparable across the three cohorts with respect to sepsis diagnosis or onset. A discussion on whether these biomarkers might be detectable prior to sepsis development would add further translational relevance.

(Remarks on code availability)
The figshare data does not include the gene name so it was hard to evaluate the code.

The code did not include data normalization and processing.

Reviewer #2

(Remarks to the Author)
This is a very interesting discovery and validation cohort(s) study seeking to identify sub-phenotypes of AKI in the setting of sepsis – and while Drs. Legrand and colleagues have done an amazing job using 3 distinct cohorts to fine tune their findings, there are several questions / issues that remain prior to publications

1) Please provide information about how common / uncommon it wwas to have a prior baseline creatinine vs. using the first SCr of the admission as baseline – as this will be important to understand and perhaps impact phenotyping process
2) The authors should clearly state how sepsis was defined across all 3 cohorts used and if there were differences etc….as they only define AKI as by the KDIGO SCr based Stage 1 definition
3) Please provide references for the ensemble clustering techniques you have used – has it been used before in AKI or nephrology critical care space?
4) 7 days from admission is a very long time – do the authors have the ability to look at sepsis-AKI that was present w/in 48 or 72 hours of admission – and those w/ later S-AKI? As S-AKI that develops on day 7 is likely VERY different than when it is present on admission or w/ the the first 24-48 hours and this may be something that is needs to be included in the clustering / modeling.? As it seems like the SCr at enrollment is different for cohorts
5) As the multiple components of MAKE90 are very different do they have data on which components were more associated w/ Phenotype 1 – was it driven by death ? or change in GFR? Continued RRT? As there are clear differences in APACHE score across individual cohorts and it would be good to know if the effects are
6) In the hundreds of protein measured did they measure Ang 2 or Ang 1 – or STNFR – which had been shown to be different in the BHatraju paper? Did they attempt to validate these other findings rather than just making a new cluster / biomarker panel
7) It seems like the there are missing sources of sepsis for the cohort in Table 1
8) Table 1 should have baseline SCr (and enrollment SCr) if these are different – we suspect they are
9) Also in table 1 – it says there are 132 in Endotype 1 – for EARLI but the calculations seemed based on 60 patietns (n%) – please corroborate that these numbers are correct as it is confusing – also the table calls them endo-types ? please harmonize
10) Is it possible to bring the outcomes data into the main paper? – as well as provide more granularity – as the report severe AKI but it is not defined in the paper ? there is no mention of RRT at the time of enrollment (or w/in 7 days) – and as above in point 5 – the breakdown of the components of MAKE
11) Revival data has MAKE90 data but not ICU free time?
12) Based on the patients enrolled and Sup table 3 – do the authors have a sense of why the rates of phenotype 1 and 2 would be 50:50% in some cohorts vs. 33:66% in EARLI ? we wonder (as above) if demographic or clinical information should be fed into a larger model that accounts for these factors AND biomarkers
13) In supplement table 4 – it seems like the authors are mixing TIMP1 and TIMP2 – they are similar but not the same
14) What happens if the authors do not take a Bayesian approach to the analyses? Is there no difference in the outcomes? Their decision to use these techniques should be more prominent in the discussion

(Remarks on code availability)

Reviewer #3

(Remarks to the Author)
Note that this review is primarily a statistical review.

Summary and overall assessment
The methods are state of the art, clearly described and correctly executed. Despite the underlying methodological complexity the paper is easy to read. The authors should be congratulated for a careful analysis and clear presentation. My overall view is that the manuscript is interesting, well written, and requires only minor revisions before publication.

Major comments
1. Methods and clarity
The statistical methods used are appropriate and current, and the authors describe implementation and validation in a way that supports reproducibility. The manuscript benefits from clear exposition that makes the advanced techniques accessible to a broad readership.

2. Generalisability to clinical roll out
A clear strength is that the data are fully comparable across the contributing studies. The manuscript should, however, discuss more explicitly whether that comparability will be preserved in a real world clinical roll out. Practical differences between research cohorts and routine clinical data (sample handling, assay platforms, pre-analytic variability, patient selection) can affect comparability and therefore performance. I recommend adding a short section or paragraph that: (a) lists the main sources of potential heterogeneity in routine practice, (b) indicates which of those were controlled for in the current pooled analysis, and (c) outlines how remaining differences might be mitigated or monitored during deployment.

3. Availability of data in routine care
The manuscript should address whether the features used in the models are expected to be widely and routinely available in everyday clinical contexts. If some predictors rely on assays or metadata that are uncommon outside specialised studies, this limits scalability. Please state which predictors are routine versus specialised, and discuss feasibility and barriers for

broad availability.

4. Timing of data acquisition relative to treatment decisions
The current analysis uses archived samples from prior studies. For clinical utility it is important to specify how timing of data collection relates to decision points in usual care. Please clarify the typical interval between sample acquisition and the treatment decisions the model is intended to inform, and discuss potential effects of timing mismatches (for example, delayed sampling, or sampling post treatment). If feasible, add a short sensitivity discussion on how predictive performance might change when measurements are taken at different clinically relevant times.

5. Stability of clustering and sample size effects
The paper reports two clusters as the principal partition. The authors should discuss the expected behaviour of clustering methods as sample size grows. In many settings the number of inferred clusters increases with larger data because estimation error decreases and more subtle structure becomes detectable. Given that the current data sets are relatively small, the manuscript should include additional analyses or commentary on cluster stability: for example, bootstrap or subsampling stability metrics including artificial enlargement of the samle size. If additional analyses are not possible within a minor revision, at minimum add a more explicit caveat in the Discussion about potential sample size dependence of the cluster count and the implications for
interpretation and clinical translation.

Minor comments and editorial suggestions
Supplementary material: indicate in the main text where readers can find code or scripts used for the primary statistical workflows to strengthen reproducibility claims.

(Remarks on code availability)
I have not previously review the underlying code as part of a review. I like the idea, but it will require so much work that it is outside of what I can offer at this time.

Version 1:

Reviewer comments:

Reviewer #1

(Remarks to the Author)
The author has addressed all my concerns.

(Remarks on code availability)

Reviewer #2

(Remarks to the Author)
The authors have sufficiently responded to my concerns and the other reviewers
nothing further from me

(Remarks on code availability)

Reviewer #3

(Remarks to the Author)
Thank you for careful consideration of my comments and for making appropriate changes to the manuscript. I have no further comments.

(Remarks on code availability)

Open Access This Peer Review File is licensed under a Creative Commons Attribution 4.0 International License, which permits use, sharing, adaptation, distribution and reproduction in any medium or format, as long as you give appropriate credit to the original author(s) and the source, provide a link to the Creative Commons license, and indicate if changes were made.
In cases where reviewers are anonymous, credit should be given to 'Anonymous Referee' and the source.
The images or other third party material in this Peer Review File are included in the article's Creative Commons license, unless indicated otherwise in a credit line to the material. If material is not included in the article's Creative Commons license and your intended use is not permitted by statutory regulation or exceeds the permitted use, you will need to obtain permission directly from the copyright holder.
To view a copy of this license, visit https://creativecommons.org/licenses/by/4.0/

We would like to thank the reviewers for their time and expertise in providing very constructive feedback and comments. We have addressed all comments and adjusted the manuscript and supplement accordingly. We hope this revised version will meet the reviewers' and editors' expectations.

Reviewer #1:

In the study by Legrand et al., the authors performed a plasma proteomics analysis on patients with sepsis-associated acute kidney injury (SA-AKI). They identified two major patient clusters that were consistently associated with sepsis-related mortality and survival across three independent clinical trials (ADRENOSS, n = 319; EARLI, n = 169; REVIVAL, n = 611). Subsequently, they developed a seven-protein predictive model to classify patients into these clusters. Notably, Bayesian modeling applied to the REVIVAL cohort suggested stronger discriminative performance among patients treated with ilofotase alfa. Overall, the findings are compelling and highlight potential biomarker targets for patient stratification and therapeutic response prediction in sepsis-associated kidney injury.

Reply: thank you for your positive and encouraging comment

Major Comments
1. The original EARLI study reportedly included 818 samples, yet only 169 were analyzed in the present work. The criteria for this subset selection remain unclear clarification is needed regarding why only approximately 20% of available samples were used.

Reply: The EARLI study originally enrolled 1,622 patients; after restricting to participants with available Olink biomarker data (due to resource limitations), acute kidney injury present, and no end-stage renal disease, 169 patients were retained for validation. We added this flow chart in the supplementary file (new eFigure 3) and modified the text page 9, line 24-27 in Manuscript.

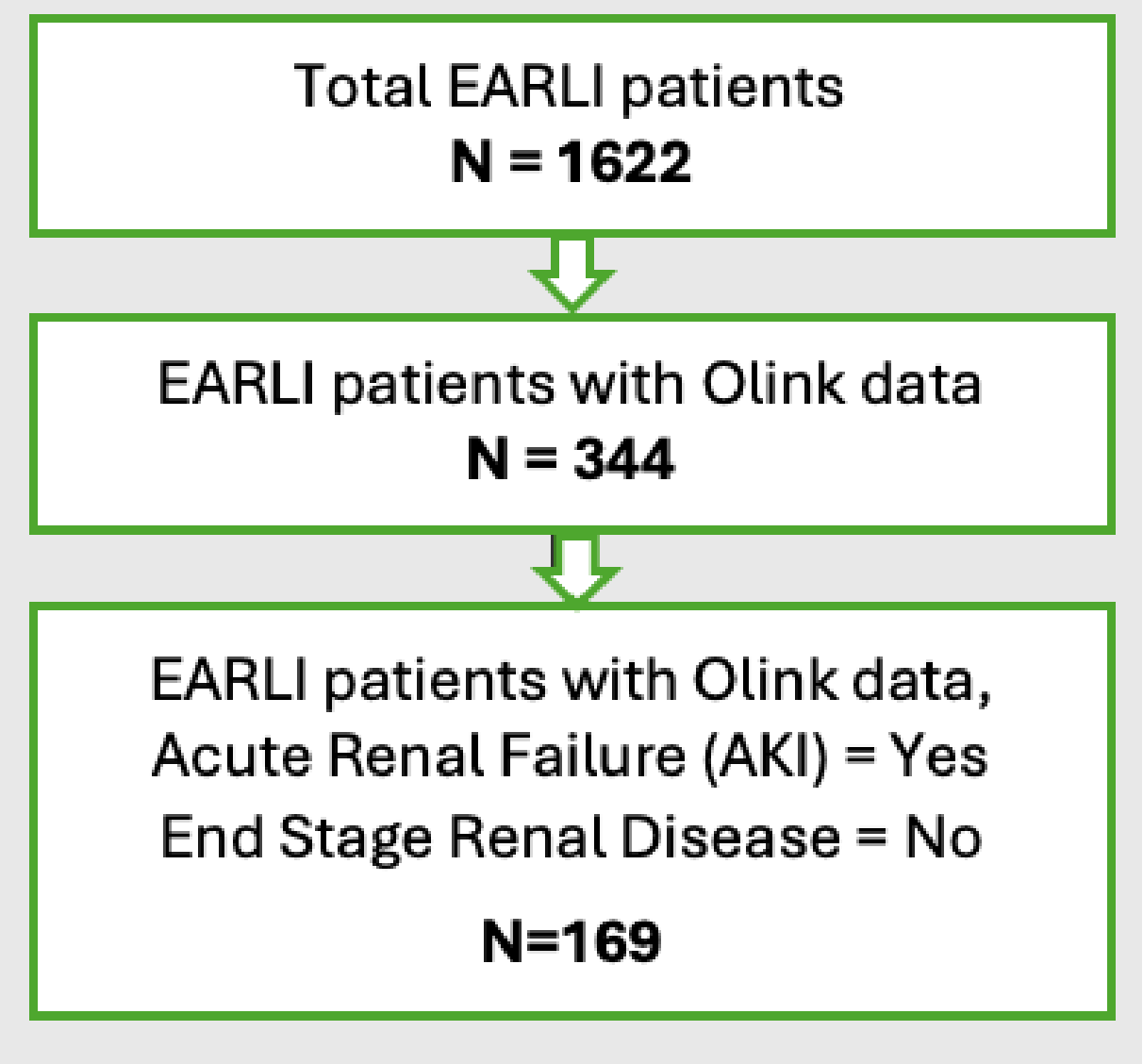

2. Given that the VALID trial cohort might be available, does the proteomics clustering and seven-protein model be validated in the VALID cohort?

Reply: Thank you for this suggestion. We do have access to the VALID cohort. Unfortunately, additional proteomic analyses in this cohort would entail substantial funding and resource expenditures which we presently are unable to do. We sincerely appreciate the reviewer's point and believe the current manuscript's inclusion of two independent validation cohorts attests to the generalizability of the model and approach. We thank the reviewer for their understanding.

3. Question on cell-of-origin of the plasma protein. Beyond pathway enrichment results, it would be valuable to explore the cell of origin and tissue sources of the seven identified proteins. Identifying which organs predominantly secrete these proteins could enhance the biological interpretability of the clustering and its relation to organ-specific pathophysiology.

Reply: We thank the reviewer for this helpful suggestion. To address this point, we performed a tissue and cell type expression analysis of the seven proteins using publicly available normal human transcriptomic data from the CELLxGENE database. These analyses are now presented in Supplementary Figure 10 with accompanying additions to the Methods, Results, and Discussion sections.

First, we examined tissue-level expression of the corresponding genes (PPP1R2, CLEC1A, LACTB2, IGFBP7, PAG1, TIMP1, and ADGRE5) across normal human tissues. IGFBP7 showed highest expression in vasculature, while the remaining genes showed highest expression in pleura. To assess coordinated expression across tissues, we calculated a composite signature score defined as the mean scaled expression across the seven genes. Ranking tissues by this score identified pleura, esophagus, vasculature, gallbladder, and musculature as the tissues with the strongest enrichment of the gene set (Supplementary Fig. 10A–C).

We next examined cell-type level expression within these five tissues. Expression was distributed across stromal, vascular, epithelial, and immune compartments, with no single lineage dominating across all tissues (Supplementary Fig. 10D). When we compared the top 20 highest scoring cell types per tissue, connective tissue cells, fibroblasts, and stromal cells appeared in four of the five tissues. Several additional populations appeared in three tissues, including endothelial cells, epithelial cells, macrophages, mesothelial cells, myeloid leukocytes, neutrophils, and smooth muscle cells (Supplementary Fig. 10E–F).

Together, these results suggest that the candidate plasma proteins may arise from a mixed cellular origin, with consistent representation from stromal and vascular populations and additional contributions from epithelial and immune lineages. We note that this analysis is based on RNA expression in normal tissues and therefore reflects baseline transcriptional patterns rather than protein secretion during

sepsis. Disease states can alter tissue programs and circulating protein levels, and RNA abundance does not directly predict protein secretion. We have added those elements in the Discussion.

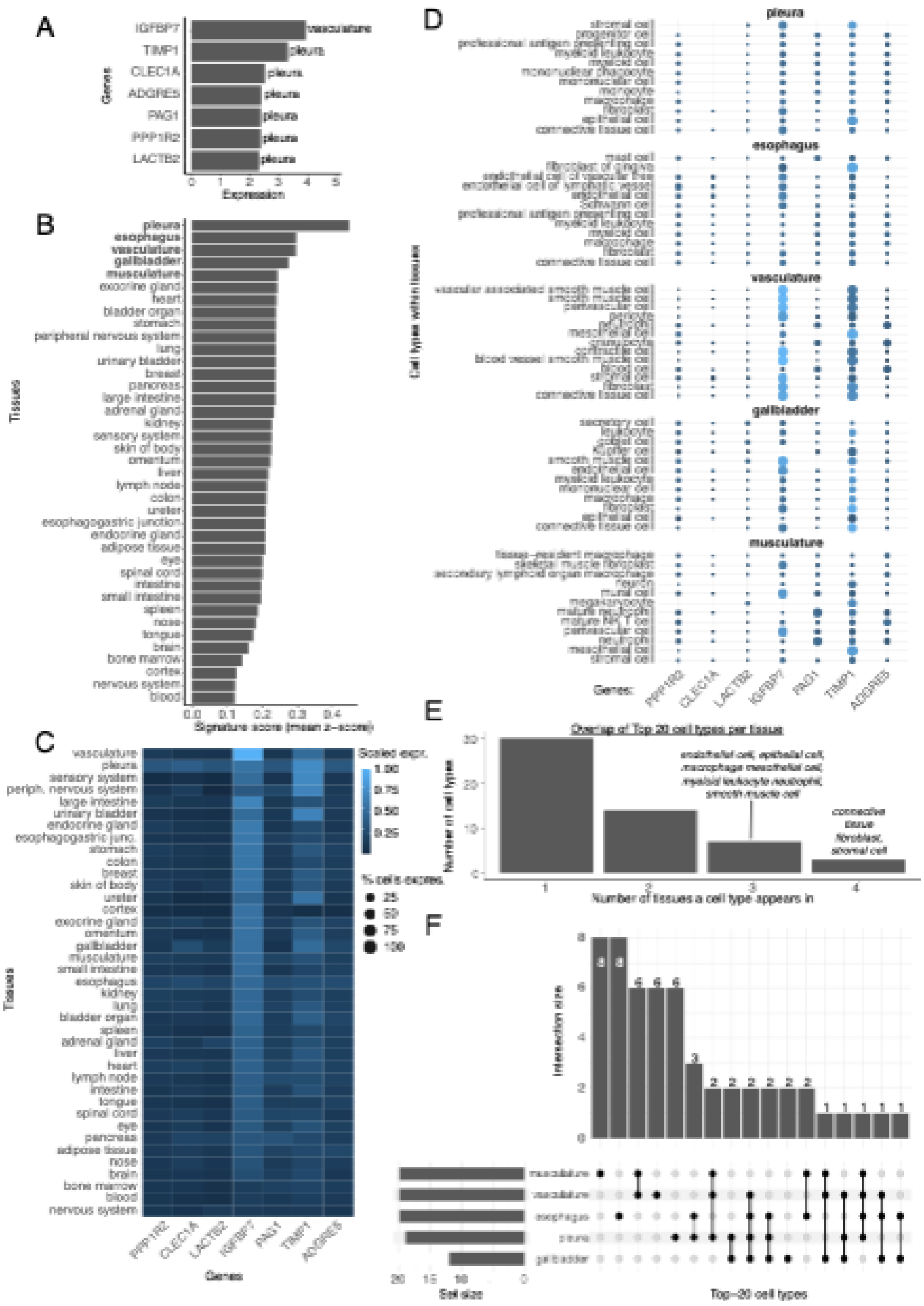

4. Question on data processing. The reviewer dataset appears to contain scaled or normalized values (including negative numbers). The manuscript should clarify the preprocessing steps applied to the proteomics data and discuss whether new patient samples can be normalized and integrated consistently into the existing model framework when predicting cluster membership.

Reply: We thank the reviewer for raising this point regarding proteomic data preprocessing. We have revised the Methods section (Biomarker measurements) to explicitly describe the use of Olink Normalized Protein eXpression (NPX) values, which are reported on a log2 scale and generated using Olink's standardized

preprocessing pipeline (including extension control, inter-plate normalization, and median normalization). We now clarify that negative NPX values may result from this normalization. We also added text to the Ensemble Clustering section stating that, because NPX values are already log-transformed and normalized by the Olink platform, no additional feature scaling or normalization was applied prior to clustering, and the same preprocessing approach was used consistently across all cohorts.

We modified page 6, lines 13-15 in the manuscript.

5. Inclusion of healthy control. As part of model validation, it would be informative to assess the seven-protein model using normal plasma samples (if available). In principle, such samples should cluster with the lower-risk group, providing an important biological sanity check for the model.

Reply: As requested by the reviewer, we now evaluated an independent cohort of healthy control samples (n = 108, drawn from publicly available data at https://www.ebi.ac.uk/pride/archive/affinity-proteomics), with biomarkers measured on the same Olink platform and standardized using ADRENOSS, and explored the classification and unsupervised clustering in healthy volunteers.

-<u>Classification</u> (models trained in ADRENOSS, predicting cluster assignment for healthy control samples):

- **Seven-biomarker model: 105/108 samples (97%) were assigned to the lower-risk subphenotype**
- **Full biomarker model: 104/108 samples (96%) were assigned to the lower-risk subphenotype**

-**<u>Unsupervised clustering</u> (ADRENOSS + healthy controls combined, n = 427):**

- **Full biomarker model: 108/108 (100%) healthy samples clustered in the lower-risk subphenotype**
- **Seven-biomarker model: 106/108 (98%) healthy samples clustered in the lower-risk subphenotype**

Overall, the vast majority of healthy samples were indeed assigned to the lower-risk subphenotype across both classification and unsupervised clustering analyses.

We modified the manuscript page 12, line 2-5 to include these results.

6. Timing of plasma collection. Since plasma samples were collected at study enrollment in each trial, it is important to clarify whether the timing of sample collection was comparable across the three cohorts with respect to sepsis diagnosis or onset. A discussion on whether these biomarkers might be detectable prior to sepsis development would add further translational relevance.

Reply: Thank you for your comment. The samples were collected at the time of meeting inclusion criteria and enrollment in the 3 cohorts. All patients had met the

criteria for sepsis at the time for sample collections. Therefore the biomarkers were measured after onset of sepsis.

Reviewer #1 (Remarks on code availability):

The figshare data does not include the gene name so it was hard to evaluate the code.

Reply: We thank the reviewer for this comment and have now attached the mapping file for UniProt and Gene Name.

The code did not include data normalization and processing.

Reply: We thank the reviewer for raising this point regarding proteomic data preprocessing. We have revised the Methods section (Biomarker measurements) to explicitly describe the use of Olink Normalized Protein eXpression (NPX) values, which are reported on a log2 scale and generated using Olink's standardized preprocessing pipeline (including extension control, inter-plate normalization, and median normalization). We now clarify that negative NPX values may result from this normalization. We also added text to the Ensemble Clustering section stating that, because NPX values are already log-transformed and normalized by the Olink platform, no additional feature scaling or normalization was applied prior to clustering, and the same preprocessing approach was used consistently across all cohorts.
We modified page 6, lines 17-19 in manuscript.

Reviewer #2 (Remarks to the Author):

This is a very interesting discovery and validation cohort(s) study seeking to identify sub-phenotypes of AKI in the setting of sepsis – and while Drs. Legrand and colleagues have done an amazing job using 3 distinct cohorts to fine tune their findings, there are several questions / issues that remain prior to publications

Reply: Thank you for your positive and encouraging comment

1) Please provide information about how common / uncommon it was to have a prior baseline creatinine vs. using the first SCr of the admission as baseline – as this will be important to understand and perhaps impact phenotyping process

Reply: We now clarify the availability and operational definition of baseline creatinine (prior to AKI) in each cohort and have updated Table 1 and in the method section as below:

- **In REVIVAL, pre-AKI creatinine values were available and was used as the baseline.**
- **In ADRENOSS, creatinine measurements prior to hospital admission were unavailable; therefore, the baseline value was determined previously per Hollinger et al (PMID: 30450469)**

- **In EARLI, baseline creatinine was defined as the most recent serum creatinine value measured within 6 months of admission, when available. Pre-admission creatinine values were available for 66 of 169 patients; for the remaining patients, the earliest creatinine value obtained after admission was used as baseline.**

We updated Table 1 to report baseline serum creatinine and pre-AKI estimated glomerular filtration rate (eGFR). For EARLI, eGFR reflects pre-admission creatinine when available and admission creatinine otherwise.

We added two rows in Table 1 in the manuscript.

2) The authors should clearly state how sepsis was defined across all 3 cohorts used and if there were differences etc….as they only define AKI as by the KDIGO SCr based Stage 1 definition

Reply: We thank the reviewer for identifying this point. Sepsis was identified per the sepsis-3 definition in the 3 cohorts. We have clarified this point in the methods section.

3) Please provide references for the ensemble clustering techniques you have used – has it been used before in AKI or nephrology critical care space?

Reply: The clustering method we used is here

19. Chiu DS, Talhouk A. diceR: an R package for class discovery using an ensemble driven approach. BMC Bioinformatics. 2018 Jan 15;19(1):11. doi: 10.1186/s12859-017-1996-y. PMID: 29334888; PMCID: PMC5769335.(https://pmc.ncbi.nlm.nih.gov/articles/PMC5769335/)

And for applications of the general class of "consensus clustering" methods in AKI / nephrology critical care applications:

16. **Thongprayoon C, Vaitla P, Nissaisorakarn V, Mao MA, Genovez JLZ, Kattah AG, Pattharanitima P, Vallabhajosyula S, Keddis MT, Qureshi F, et al. Clinically Distinct Subtypes of Acute Kidney Injury on Hospital Admission Identified by Machine Learning Consensus Clustering. Medical Sciences. 2021; 9(4):60. https://doi.org/10.3390/medsci9040060 (https://www.mdpi.com/2076-3271/9/4/60)**
17. **Pattharanitima P, Thongprayoon C, Petnak T, Srivali N, Gembillo G, Kaewput W, Chesdachai S, Vallabhajosyula S, O'Corragain OA, Mao MA, Garovic VD, Qureshi F, Dillon JJ, Cheungpasitporn W. Machine Learning Consensus Clustering Approach for Patients with Lactic Acidosis in Intensive Care Units. J Pers Med. 2021 Nov 2;11(11):1132. doi: 10.3390/jpm11111132. PMID: 34834484; PMCID: PMC8623582.**
18. **Pan HC, Sun CY, Huang TT, Huang CT, Tsao CH, Lai CH, Chen YM, Wu VC. Distinct Subtyping of Successful Weaning from Acute Kidney Injury Requiring Renal Replacement Therapy by Consensus Clustering in Critically Ill Patients.**

Biomedicines. 2022 Jul 7;10(7):1628. doi: 10.3390/biomedicines10071628. PMID: 35884933; PMCID: PMC9313082.

We updated the references list accordingly.

4) 7 days from admission is a very long time – do the authors have the ability to look at sepsis-AKI that was present w/in 48 or 72 hours of admission – and those w/ later S-AKI? As S-AKI that develops on day 7 is likely VERY different than when it is present on admission or w/ the the first 24-48 hours and this may be something that is needs to be included in the clustering / modeling.? As it seems like the SCr at enrollment is different for cohorts

Reply: We agree with the reviewer that this is an important issue and performed additional analyses.
AKI within 48 hours was an inclusion criterion for REVIVAL, in the other 2 cohorts AKI could be present for longer than 48 hours. So the full study qualified for both the AKI within 48 hours and AKI within 7-day criteria.
In the 2 other cohorts, we evaluated the effect of reducing our analysis cohort to patients meeting AKI criteria within 48 hours of admission (EARLI n=76; ADRENOSS n=216). As a sensitivity analysis, we repeated clustering and downstream biomarker analyses using the 48-hour S-AKI definition in ADRENOSS and EARLI. We also compared the rankings of biomarker importance between the original 7-day model and the 48-hour model.
Below are the clustering results:

ADRENOSS:

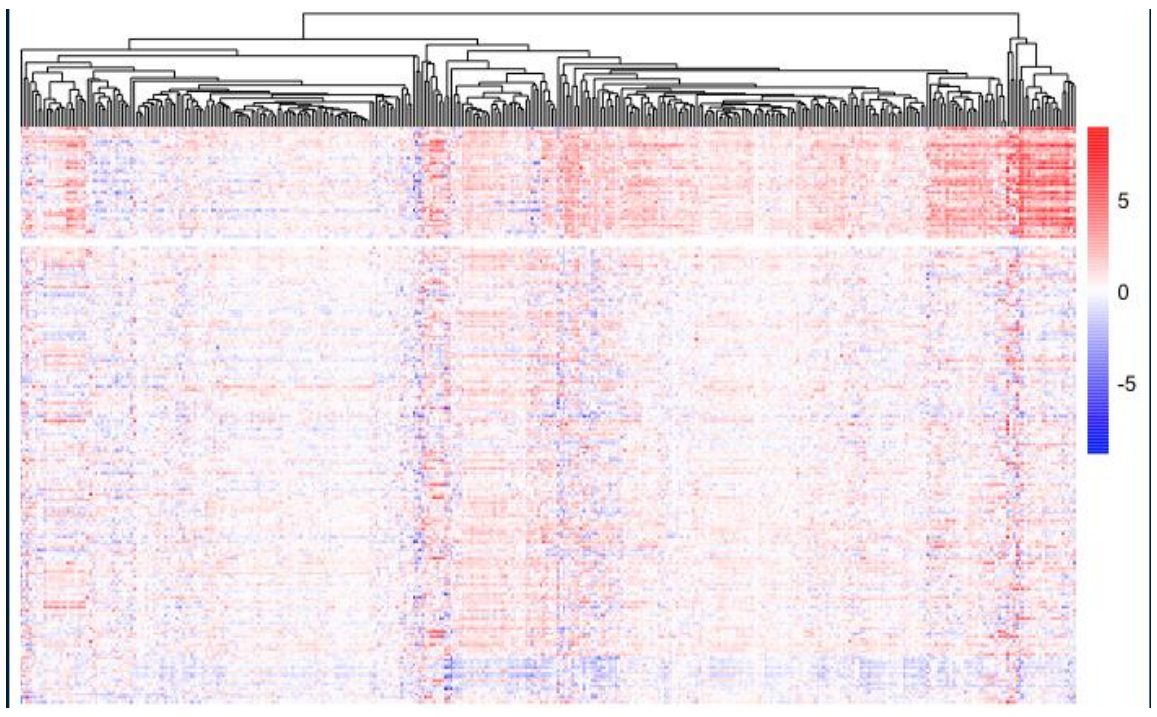

Outcome	1<br>N = 42[1]	2<br>N = 174[1]
MAKE.7d	90%	55%
severeAKI.7d	74%	27%
death.28d	62%	30%
death.90d	69%	40%
MAKE.90d	93%	84%

[1] %

EARLI:

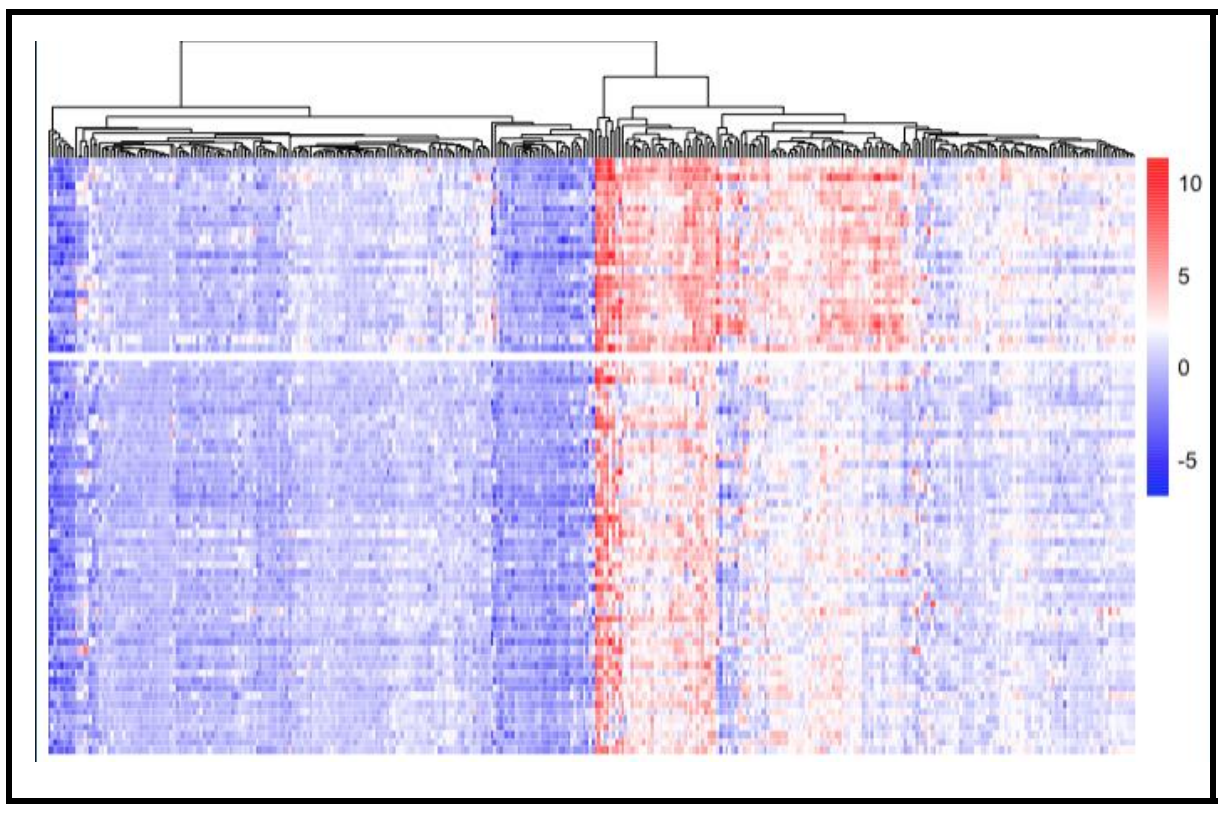

Outcome	1<br>N = 25[1]	2<br>N = 51[1]
death.28d	64%	31%
death.90d	68%	31%
MAKE.90d	76%	45%

[1] %

The correlation plots below show protein importance ranks from the 7-day model on the x-axis and from the 48-hour model on the y-axis, and we calculated rank agreement using Spearman correlation for each cohort. A high Spearman correlation indicates that biomarkers ranked as more important in one model tend to also be ranked as more important in the other model.

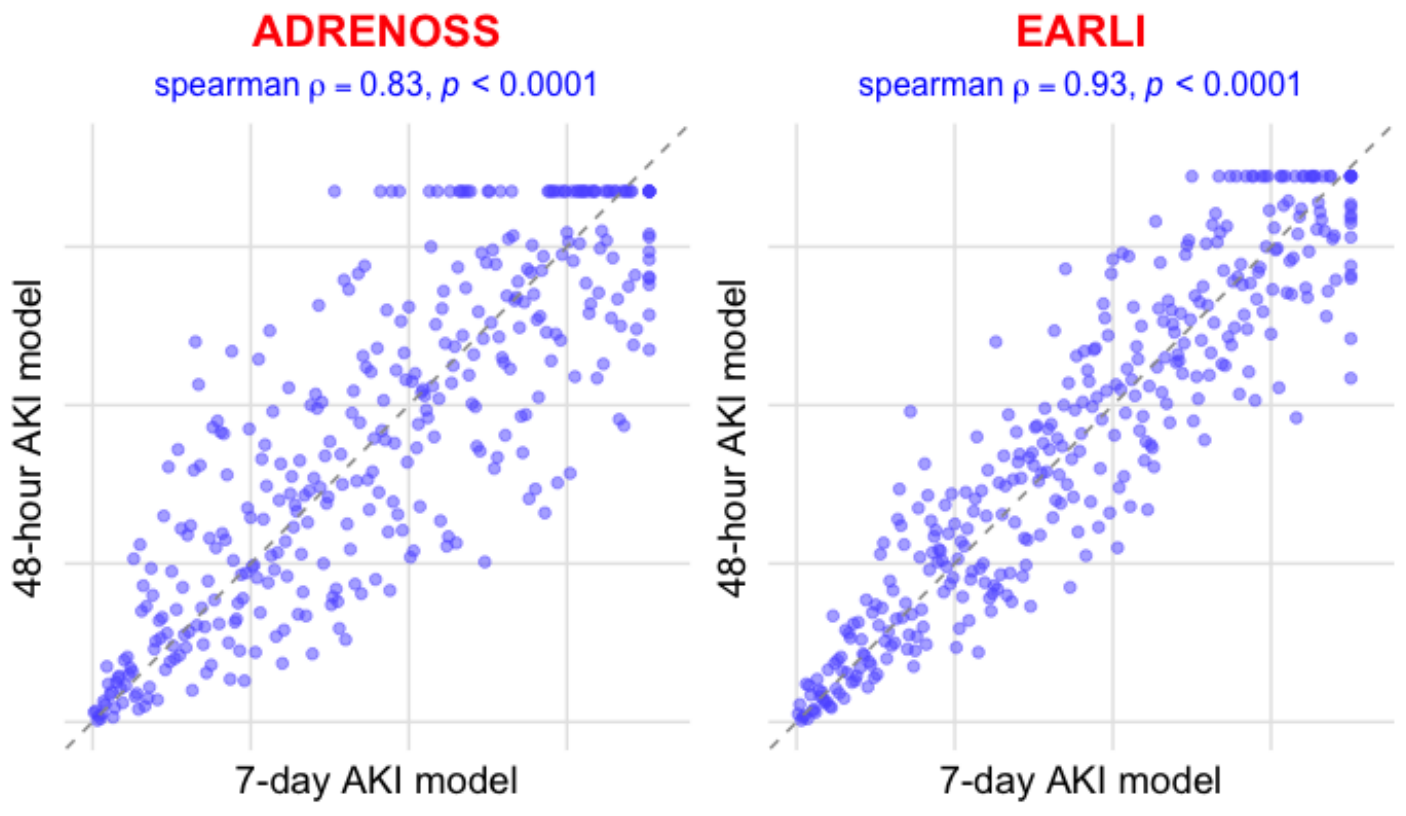

Based on these analyses, we modified the manuscript, added a sentence to page 10, line 6-7
We modified the supplement: added a sensitivity analysis section to page 8, lines 4-16.

5) As the multiple components of MAKE90 are very different do they have data on which components were more associated w/ Phenotype 1 – was it driven by death? or change in GFR? Continued RRT? As there are clear differences in APACHE score across individual cohorts and it would be good to know if the effects are

Reply: Thank you for this comment. We have added the details for the different components of MAKE in the new Supplementary Table 7. Please note that the total exceeds 100 % since patients may meet several criteria (for instance needing RRT and then died).

Supplementary Table 7: Detailed MAKE90 components across three cohorts. Supplement page 17.

6) In the hundreds of proteins measured, did they measure Ang 2 or Ang 1 – or STNFR – which had been shown to be different in the BHatraju paper? Did they attempt to validate these other findings rather than just making a new cluster / biomarker panel

Reply: Thank you for this important comment. We examined Ang1, Ang2, and sTNFR1 in the EARLI cohort. Among 169 EARLI patients, 108 had available measurements for all three biomarkers.

Following the approach of Bhatraju et al., we fit two logistic regression models using the Ang2/Ang1 ratio and sTNFR1 as predictors:
(a) a model with the full-model cluster assignment as the outcome, and (b) a model with the parsimonious cluster assignment as the outcome. Model discrimination was assessed as an area under the curve (AUC) for a ROC plot. The resulting AUCs of 0.669 and 0.655 for the two models (see Figures below), respectively, suggest limited ability to detect our cluster assignments. These data are consistent with the clusters we identify as being distinct from and are not robustly aligned with the Ang1/Ang2-based cluster structure.

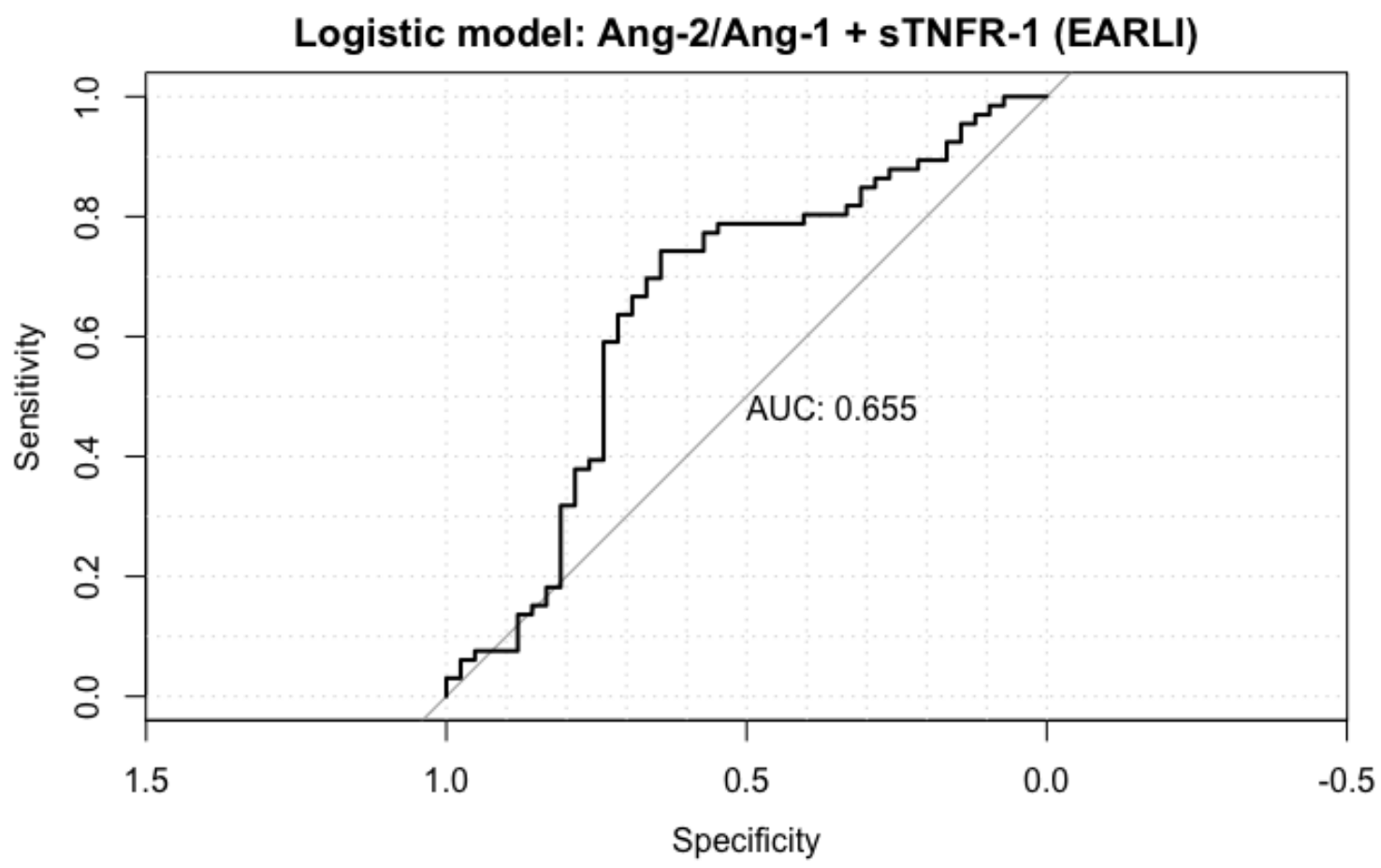

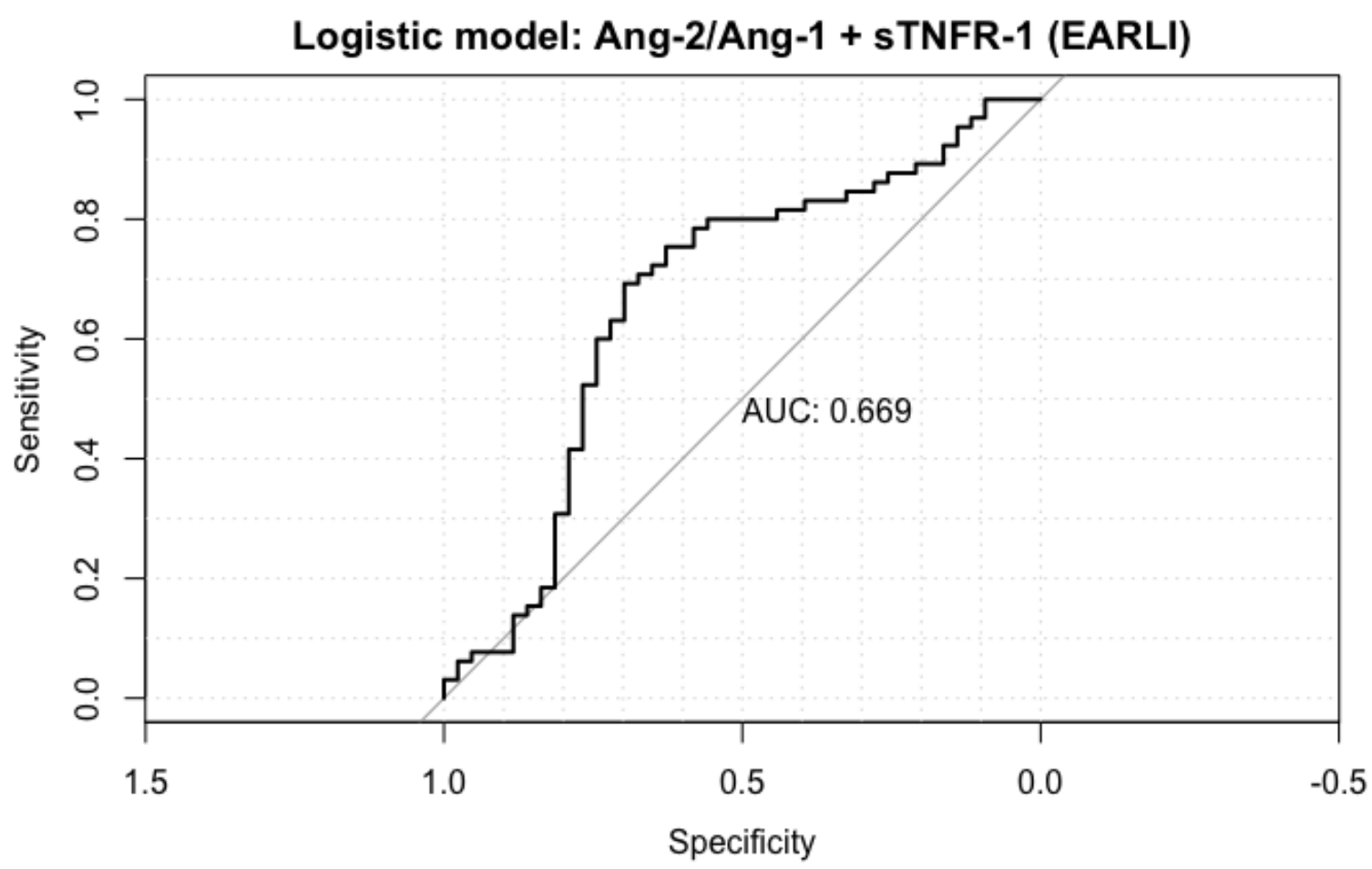

We also compared clustering based on Ang1, Ang2, and sTNFR1 with our parsimonious clusters and found that the three gene-derived clusters are distinct from ours: Jaccard = 0.32 and ARI = 0.15.

We modified page 15, lines 1-6 in the manuscript.
We modified lines 117-20 on page 8, and 1-7 on page 9 of the supplement.

7) It seems like the there are missing sources of sepsis for the cohort in Table 1

Reply: Thank you, we have updated the names from Sepsis Origin to Sources of Sepsis.

8) Table 1 should have baseline SCr (and enrollment SCr) if these are different – we suspect they are

Reply: Thank you, we have updated Table 1 to reflect this information.

9) Also in table 1 – it says there are 132 in Endotype 1 – for EARLI but the calculations seemed based on 60 patietns (n%) – please corroborate that these numbers are correct as it is confusing – also the table calls them endo-types? please harmonize

Reply: Thank you for catching those discrepancies. We have corrected the numbers and the terminology.

10) – as well as provide more granularity – as the report severe AKI but it is not defined in the paper ? there is no mention of RRT at the time of enrollment (or w/in 7 days) – and as above in point 5 – the breakdown of the components of MAKE

Reply: Yes, we can describe the outcomes data in the main paper. Thank you for this comment. We have added the details for the different components of MAKE in the new Supplementary Table 7. Please note that the total exceeds 100 % since patients may meet several criteria (for instance needing RRT and then died).

Supplementary Table 7: Detailed MAKE90 components across three cohorts:
Table 7 is updated in the supplement, page 17.

11) Revival data has MAKE90 data but not ICU free time?

Reply: Thank you, we have added ICU-free days up to 90 days to the outcome table and moved the outcome table to the manuscript.

Table 2 in manuscript, page 23.

12) Based on the patients enrolled and Sup table 3 – do the authors have a sense of why the rates of phenotype 1 and 2 would be 50:50% in some cohorts vs. 33:66% in EARLI ? we wonder (as above) if demographic or clinical information should be fed into a larger model that accounts for these factors AND biomarkers

Reply: Thank you for your comment. Regarding the rates of phenotypes across cohorts, we believe the difference between EARLI, and the other cohorts mostly lies in slightly different populations enrolled and timing of enrollment. ADRENOSS and REVIVAL enrolled patients in the ICU while EARLI could enroll patients in the emergency department. It is possible that some patients were enrolled earlier in the course of their sepsis, which may have impacted the proportion of patients with phenotype 1. We have added this hypothesis in the discussion.
Regarding the question on clinical variables, we have addressed your comments in 3 ways :

a) **We explored whether adding clinical variables to biomarkers identify different clusters:**
   We reran clustering within each cohort after incorporating clinical variables (age, sex, and comorbidities) alongside biomarkers and compared the resulting cluster assignments with those from biomarker-only analyses.
   - **When clinical variables were added to the parsimonious biomarker panel, cluster agreement with the original parsimonious clusters was high (Jaccard index: ADRENOSS = 0.87; EARLI = 0.88; REVIVAL = 0.80).**
   - **Similarly, when clinical variables were added to the full 366-biomarker panel, the resulting clusters remained comparable to the original full biomarker-only clusters (Jaccard index: ADRENOSS = 0.80; EARLI = 0.80; REVIVAL = 0.82).**

b) **We explored whether clinical variables predict our current clusters (identified using biomarkers only) :**
   We fit logistic regression models with clinical variables as covariates to predict cluster assignments derived from both the parsimonious and full biomarker models. The resulting area under the receiver operating characteristic curve (AUC) values for the parsimonious model were 0.68 for ADRENOSS, 0.78 for EARLI, and 0.64 for REVIVAL; corresponding AUC values for the full biomarker model were 0.67, 0.79, and 0.65, respectively.

c) **we explored whether adding clinical variables to biomarkers predict our current clusters better than using biomarker predictors only :**
   We used the same classification framework as in the primary analysis. Models were trained in ADRENOSS using the 7-biomarker parsimonious panel either alone or in combination with clinical variables and then applied to predict cluster assignments in the EARLI and REVIVAL cohorts. Predictive performance was comparable between models, with AUCs of 0.995 and 0.984 for the biomarker-only model in EARLI and REVIVAL, respectively, and 0.995 and 0.984 for the biomarker-plus-clinical model, indicating no meaningful improvement with the addition of clinical variables.

We modified manuscript page 14, line 6-7.
We modified the supplement page 9, lines 9-19, and page 10, lines 1-7.

13) In supplement table 4 – it seems like the authors are mixing TIMP1 and TIMP2 – they are similar but not the same

Reply: We apologize for this and have corrected this typo.

14) What happens if the authors do not take a Bayesian approach to the analyses? Is there no difference in the outcomes? Their decision to use these techniques should be more prominent in the discussion.

Reply: We have added this information in the supplementary file. of note, using a frequentist approach yields a low power to show statistical difference between groups and didn't result in a significant difference. We have added this point in the limitation section of the manuscript.

1. **Treatment effect within each cluster** (separate Cox models)

- Cluster 1: HR(recAP vs Placebo) = **0.87** (95% CI 0.60–1.26), p=0.47

- Cluster 2: HR(recAP vs Placebo) = **1.15** (95% CI 0.77–1.72), p=0.48

2. **Interaction model (treatment × cluster)**

- Interaction HR = HR of cluster 2 / HR of cluster 1 = **1.33**, p=0.30

  Treatment is less beneficial in Cluster 2 than in Cluster 1, but the p-value is not significant.

We modified the supplementary page 11, lines 1-5.

Reviewer #3 (Remarks to the Author):

Note that this review is primarily a statistical review.

Summary and overall assessment
The methods are state of the art, clearly described, and correctly executed. Despite the underlying methodological complexity, the paper is easy to read. The authors should be congratulated for a careful analysis and clear presentation. My overall view is that the manuscript is interesting, well-written, and requires only minor revisions before publication.

Reply: thank you for your positive and encouraging comment

Major comments
1. Methods and clarity
The statistical methods used are appropriate and current, and the authors describe implementation and validation in a way that supports reproducibility. The manuscript

benefits from clear exposition that makes the advanced techniques accessible to a broad readership.

Reply: We thank the reviewer for their kind and constructive comments.

2. Generalisability to clinical roll out

A clear strength is that the data are fully comparable across the contributing studies. The manuscript should, however, discuss more explicitly whether that comparability will be preserved in a real-world clinical rollout. Practical differences between research cohorts and routine clinical data (sample handling, assay platforms, pre-analytic variability, patient selection) can affect comparability and therefore performance. I recommend adding a short section or paragraph that: (a) lists the main sources of potential heterogeneity in routine practice, (b) indicates which of those were controlled for in the current pooled analysis, and (c) outlines how remaining differences might be mitigated or monitored during deployment.

Reply: We appreciate this important and constructive comment. We have added text to the manuscript that specifically details main sources of potential heterogeneity in clinical practice and how they were controlled in our pooled analysis. We also detail how remaining variance could be mitigated or monitored during deployment.

Manuscript Addition:
The model was developed using features from primary sepsis data. While this approach tacks our model to clinical sepsis, it is important to note that heterogeneity in regard to hospital assay platforms, patient comorbidities, sepsis presentation, and sample handling can all impact the fidelity of the model in predicting SAKI at the level of individual patients. During model development, these factors were controlled in part by using an ensemble approach which reduces sensitivity to stochasticity. Additionally, differences across cohorts were further examined through pooled ensemble clustering across ADRENOSS, EARLI, and REVIVAL (n=1,099). In the pooled analysis, the two-subphenotype structure was supported by the lowest Proportion of Ambiguous Clustering (PAC), indicating stability of the clustering structure across cohorts. Remaining variance arising from clinical heterogeneity could be mitigated by ensuring a standardized normalization approach to all input variables and the utilization of anomaly prediction models to discount data whose variance would translate into low confidence predictions. As more training data becomes available, periodic model retraining after deployment would also contribute to a reduction in variance.

We modified manuscript page 16, line 5-18.

3. Availability of data in routine care

The manuscript should address whether the features used in the models are expected to be widely and routinely available in everyday clinical contexts. If some predictors rely on assays or metadata that are uncommon outside specialised studies, this limits scalability. Please state which predictors are routine versus specialised, and discuss feasibility and barriers for broad availability.

Reply: We appreciate the reviewer's pertinent point about feature availability in the clinical setting. We have added text to the manuscript that discusses availability of predictors in the clinical setting and the barriers for broader availability.

Manuscript Addition:
Notably, the 7-feature parsimonious model relies on biomarkers that are measurable but not routinely available in most institutions (IGFBP-7 is commercially available and FDA-cleared). A key barrier to its clinical usability is therefore the capacity of a laboratory to assay these biomarkers. The utility of this model could potentially be enhanced by the development of a combined assay capable of measuring all 7 biomarkers simultaneously.

We modified manuscript page 16, line 26-30.

4. Timing of data acquisition relative to treatment decisions
The current analysis uses archived samples from prior studies. For clinical utility it is important to specify how timing of data collection relates to decision points in usual care. Please clarify the typical interval between sample acquisition and the treatment decisions the model is intended to inform, and discuss potential effects of timing mismatches (for example, delayed sampling, or sampling post treatment). If feasible, add a short sensitivity discussion on how predictive performance might change when measurements are taken at different clinically relevant times.

Reply: We appreciate the reviewer's point regarding the relationship between the timing of sample acquisition and treatment decisions. The model can predict patients most at risk for adverse outcomes associated with SAKI at early (24–48 hours) and late (up to 7 days) time points during sepsis. As shown in the analysis below, predictions at 48 hours and 7 days are highly correlated. Sample acquisition has the opportunity to inform treatment decisions, as initiation of treatment or supportive care occurs within this time frame in patients with sepsis (for instance, in the REVIVAL trial, treatment had to be administered within 72 hours of SAKI onset).

5. Stability of clustering and sample size effects

The paper reports two clusters as the principal partition. The authors should discuss the expected behaviour of clustering methods as the sample size grows. In many settings, the number of inferred clusters increases with larger data because estimation error decreases and a more subtle structure becomes detectable. Given that the current data sets are relatively small, the manuscript should include additional analyses or commentary on cluster stability: for example, bootstrap or subsampling stability metrics including artificial enlargement of the sample size. If additional analyses are not possible within a minor revision, at a minimum, add a more explicit caveat in the Discussion about potential sample size dependence of the cluster count and the implications for interpretation and clinical translation.

Reply: To evaluate cluster stability and potential sample size effects, we assessed stability using a resampling-based consensus clustering framework (diceR). For

each cohort separately, we evaluated cluster solutions ranging from k=2 to k=6, and quantified stability using the Proportion of Ambiguous Clustering (PAC), where lower values indicate greater partition stability.
In all three cohorts, the two-cluster solution yielded the lowest PAC, indicating the most stable partition under resampling. To further examine whether cluster number was sensitive to sample size, we repeated the consensus clustering procedure using pooled data from ADRENOSS (n=319), EARLI (n=169), and REVIVAL (n=611), yielding a combined dataset of 1,099 patients. In this enlarged dataset, the two-cluster solution again demonstrated the lowest PAC across evaluated values of k.
These findings suggest that the two-subphenotype structure is stable under resampling and is not materially altered by moderate increases in sample size within the range studied.

We modified manuscript page 16, lines 5-18.

We modified the supplementary page 9, lines 13-21

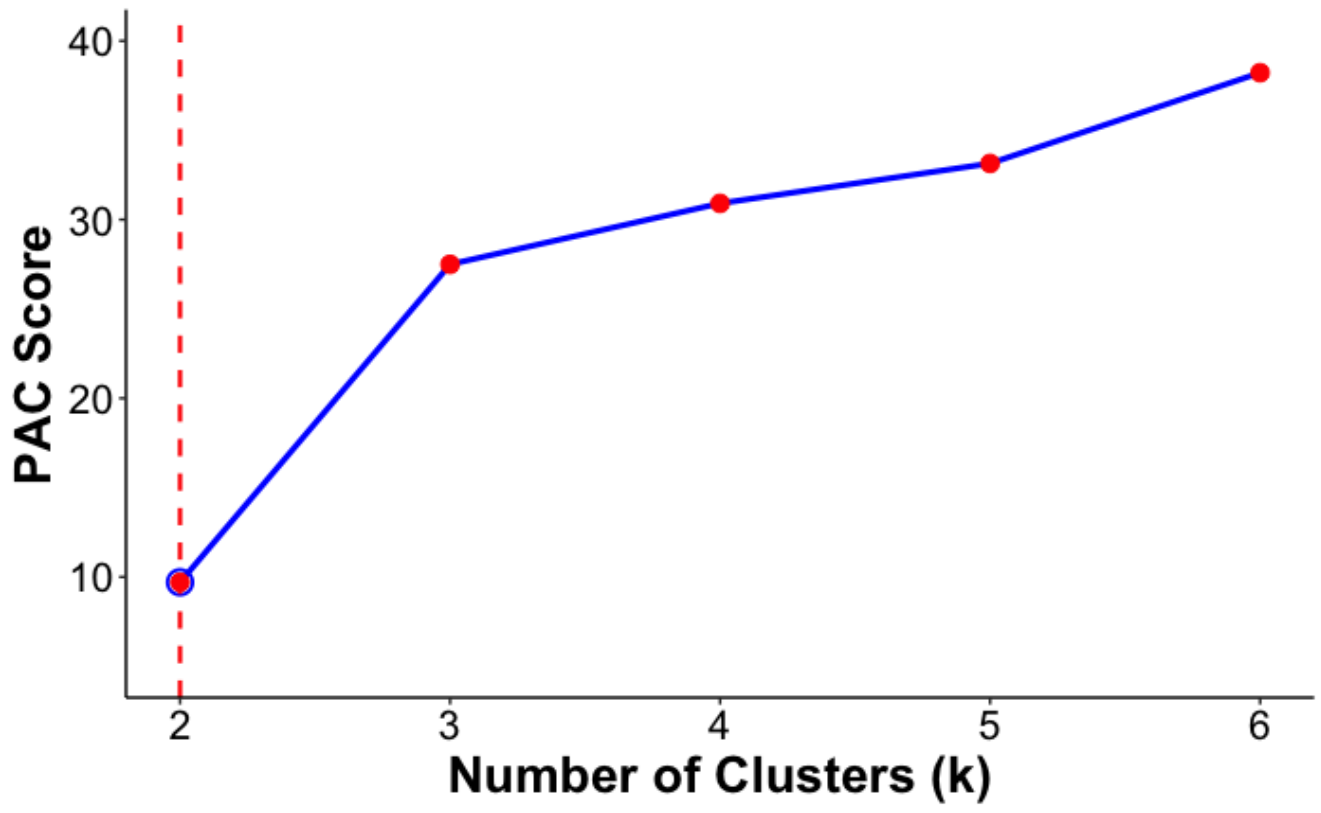

Minor comments and editorial suggestions
Supplementary material: indicate in the main text where readers can find code or scripts used for the primary statistical workflows to strengthen reproducibility claims.

Reply: we added the link in the supplement to the publicly available codes. Below is the shared GitHub repository for the project.
https://github.com/hanhnguyen24/SepsisAKI_Subphenotypes

Reviewer #3 (Remarks on code availability):

I have not previously review the underlying code as part of a review. I like the idea, but it will require so much work that it is outside of what I can offer at this time.
